# Supplementary material for: Analysis of PD1, LAG3, TIGIT, and TIM3 expression in human lung adenocarcinoma reveals a 25-gene signature predicting immunotherapy response
Source: Cell Rep Med. 2024 Nov 25;5(12):101831. doi: 10.1016/j.xcrm.2024.101831 (PMC11722093; doi:10.1016/j.xcrm.2024.101831)
Supplement: Document S1. Figures S1–S5 and Tables S1–S3 [file mmc1.pdf]

Cell Reports Medicine, Volume *5*

Supplemental information

Analysis of PD1, LAG3, TIGIT, and TIM3 expression in human lung adenocarcinoma reveals a 25-gene signature predicting immunotherapy response

Jean-Philippe Guégan, Florent Peyraud, Bérengère Dadone-Montaudie, Diego Teyssonneau, Lola-Jade Palmieri, Emma Clot, Sophie Cousin, Guilhem Roubaud, Mathilde Cabart, Laura Leroy, Coriolan Lebreton, Christophe Rey, Oren Lara, Ophélie Odin, Maxime Brunet, Lucile Vanhersecke, Ezogelin Oflazoglu Gruyters, Ikbel Achour, Leila Belcaid, Sylvestre Le Moulec, Thomas Grellety, Alban Bessede, and Antoine Italiano

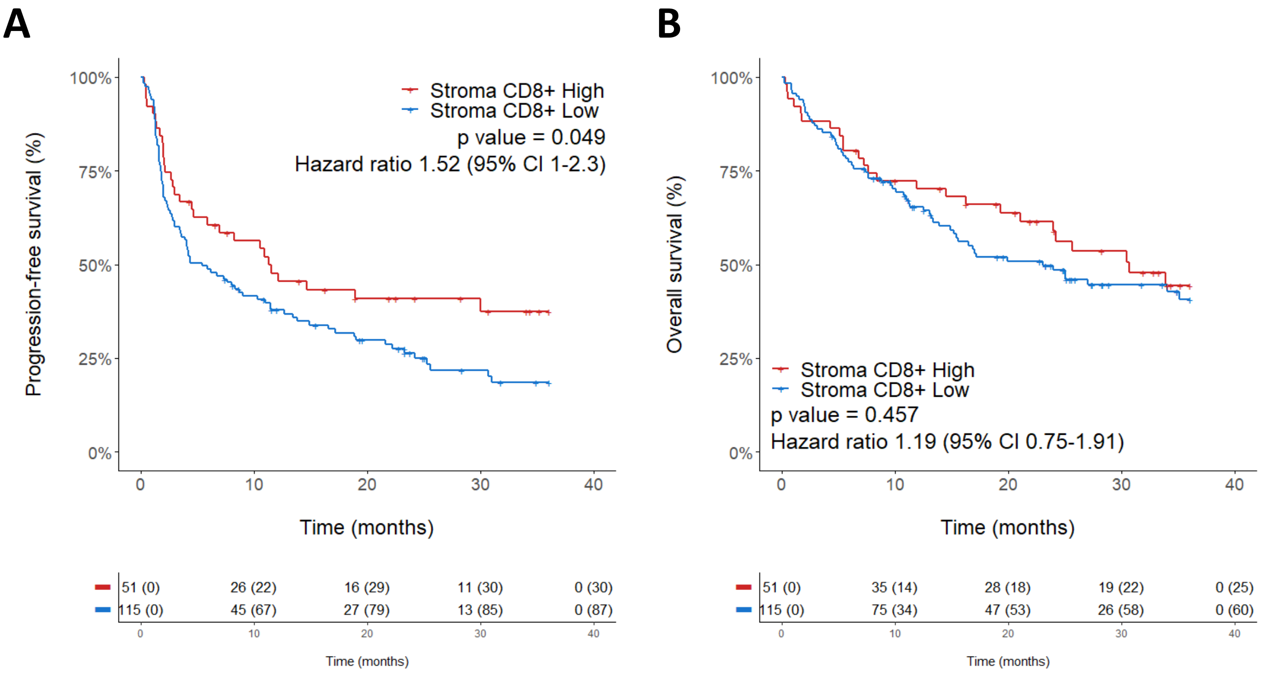

Figure S1. Impact of stromal CD8+ cells on ICI response. Related to Figure 1. Kaplan-Meier curves of progression-free survival (A) and overall survival (B) of LUAD patients with treated with ICI according to level of stroma-infiltrating CD8+ cells. Statistical significance was determined by log-rank test.

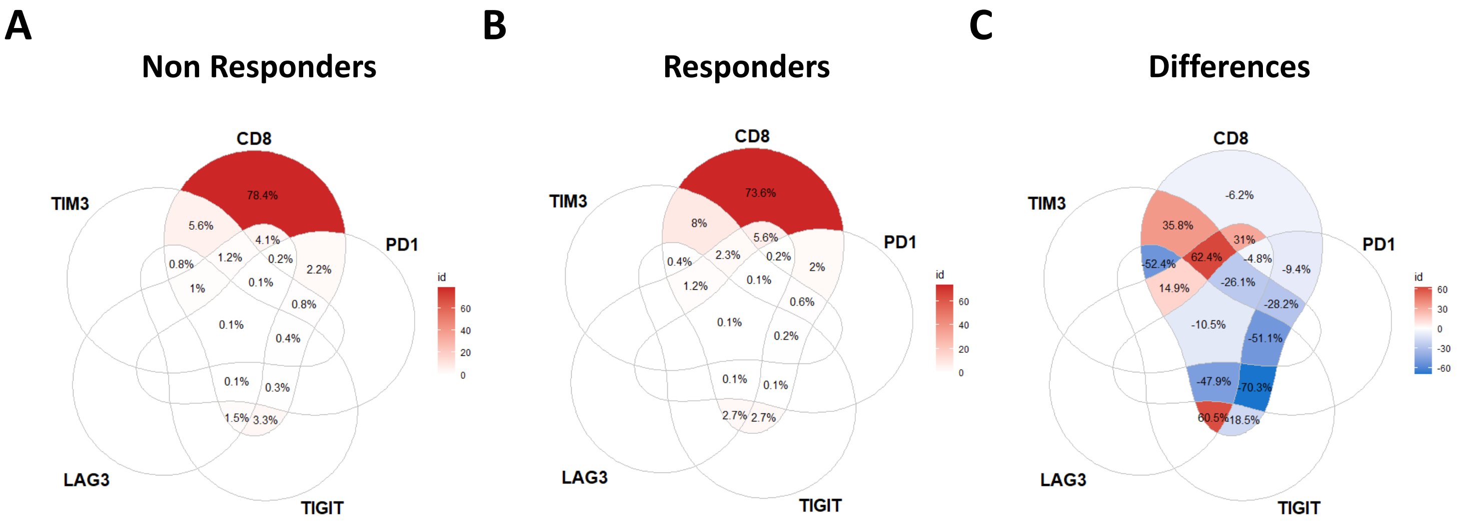

Figure S2. PD1 expression by CD8+ cells correlates with immunotherapy resistance. Related to Figure 1. **(A-C)** Venn diagrams of the expression of exhaustion markers by CD8+ cells from non-responders (A) and responders (B) patients. Differences between responders and non-responders CD8+ cells are displayed in (C). For the analysis, cohort (n=166 patients) was down-sampled to 1000 CD8+ cells per patient. Median of cell distribution are shown.

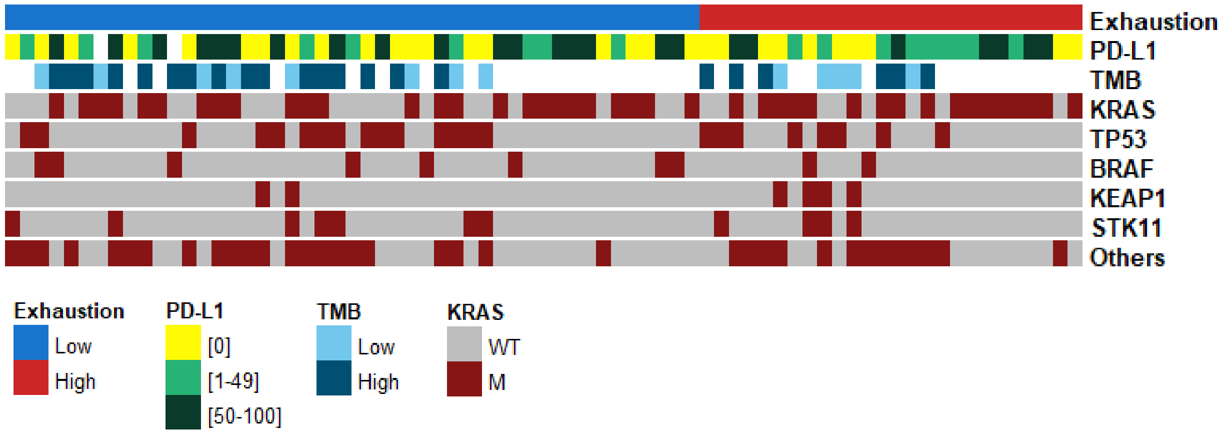

Figure S3. Mutational landscape of LUAD patients treated with ICI according to exhaustion level of CD8+ cells. Related to Figure 1. TMB stands for Tumor Mutational Burden.

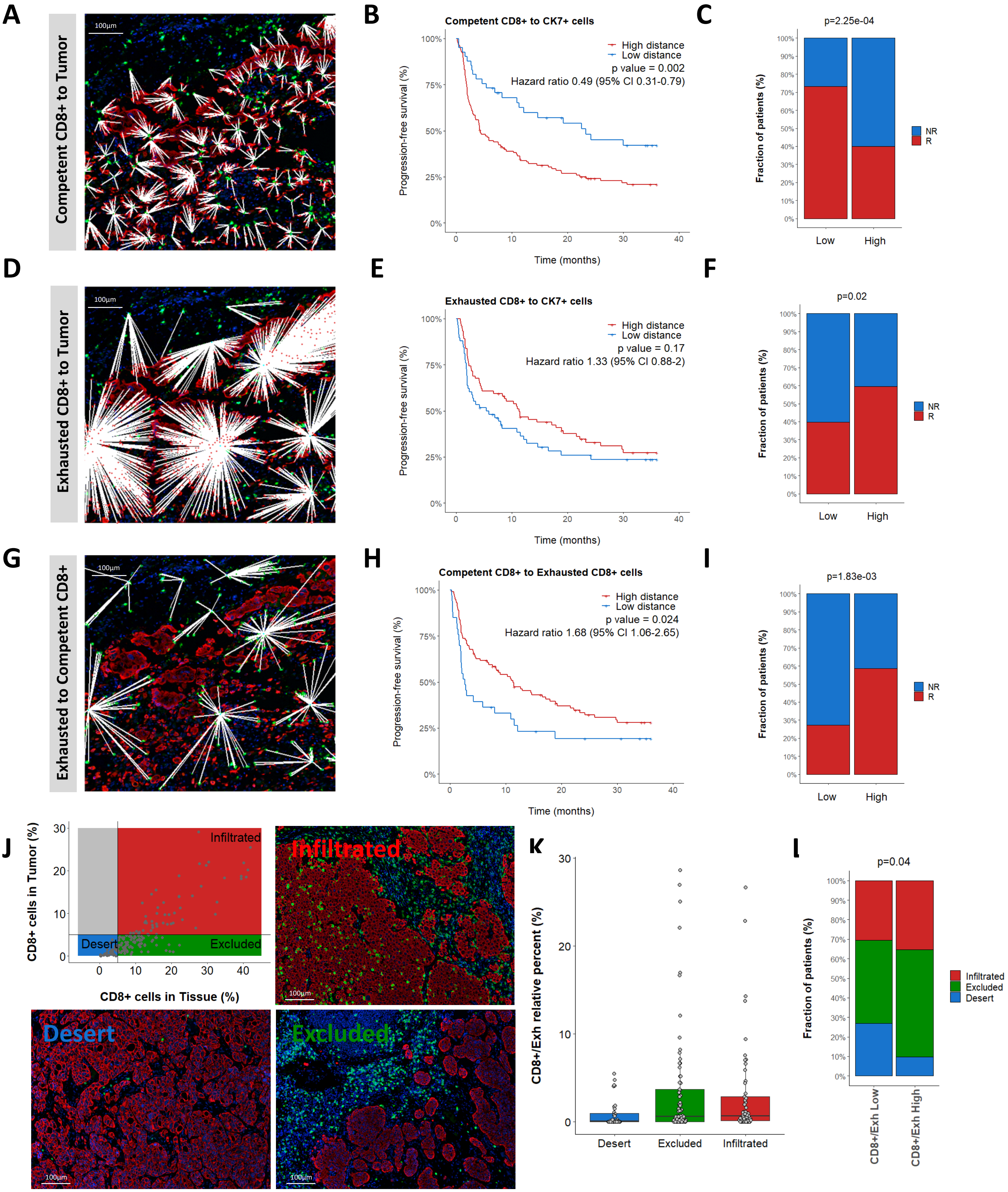

Figure S4. Spatial analysis of the CD8+ distribution in LUAD patients. Related to Figure 1. Intercellular distances were computed between "Competent" CD8+ (CD8+/PD1-), "Exhausted" CD8+ (CD8+/PD1+ ± LAG3/TIGIT/TIM3+) and tumor (CK7+) cells. (**A-D-G**) Illustration of the calculated distances between indicated cells. (**B-E-H**) Kaplan-Meier curves of progression-free survival of LUAD patients classified as "High" or "Low" based on calculated distances. Statistical significance was determined by log-rank test. (**C-F-I**) Histograms of the clinical response achieved in patients with « High » versus « Low » intercellular distances. Statistical significance was determined by chi-squared test. (**J**) Illustration of Immune contexture definition in LUAD patients. A "Desert" phenotype is considered when the sample exhibits less than 5% of CD8+ in the whole section. An "Excluded" phenotype is defined by less than 5% of CD8+ in the tumor areas of the sample. Otherwise, the sample is considered as "Infiltrated" (**K**) Boxplot representation (median ± interquartiles) of CD8+ exhaustion according to immune contexture. (**L**) Histograms of the immune contexture achieved in patients with « High » versus « Low » CD8+ exhaustion. Statistical significance was determined by chi-squared test.

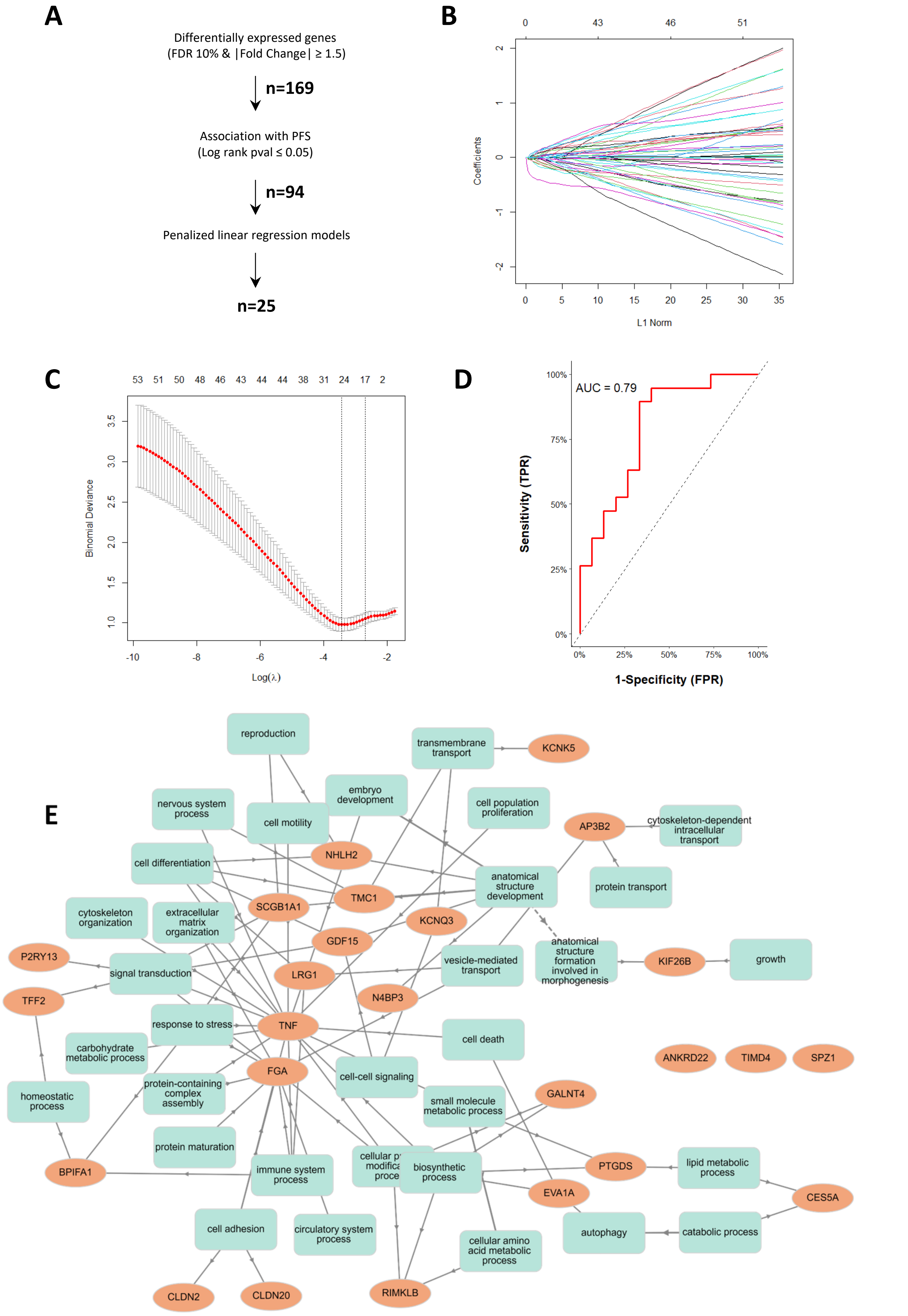

Figure S5. Establishment of a gene signature for the prediction of CD8+ exhaustion. Related to Figure 2. (**A**) Gene signature building strategy (**B**) Coefficient profile plot of the fitted glmnet model (**C**) Cross-validation curve (**D**) ROC curve for the prediction of CD8+ exhaustion in validation cohort (n=35 patients). (**E**) Relationship mapping of signature predictors to Gene Ontology terms.

Age	
≤65	89 (54)
>65	77 (46)
**Gender**	
Female	56 (34)
Male	110 (66)
**TPS**	
PD-L1 <1%	64 (39)
PD-L1 1-49%	53 (32)
PD-L1 ≥50%	47 (28)
NA	2 (1)
**ECOG PS**	
0-1	127 (77)
>1	39 (23)
**Immunotherapy**	
Anti-PD1	136 (82)
Anti-PD-L1	23 (14)
Combination**	7 (4)
**Response**	
PD	63 (38)
SD	23 (14)
OR	80 (48)
**Total**	166

*ECOG PS: Eastern Cooperative Oncology Group Performance Status; OR: overall response (partial response or complete response); PD: progressive disease; SD: stable disease; TPS: tumor proportion score.
** Combination of Immunotherapy (Durvalumab-Tremelimumab, Pembrolizumab-Feladilimab, Atzeolizumab-Bevacizumab, Atezolizumab-Daratumumab)

Supplementary Table 1. Clinical characteristics of patients. Related to Figure 1

	CD8+/Exh. High	CD8+/Exh. Low
**Age**		
≤65	27 (53)	62 (54)
>65	24 (47)	53 (46)
**Gender**		
Female	18 (35)	38 (33)
Male	33 (65)	77 (67)
**TPS**		
PD-L1 <1%	19 (37)	45 (39)
PD-L1 1-49%	17 (33)	36 (31)
PD-L1 ≥50%	15 (29)	32 (28)
NA	0 (0)	2 (2)
**ECOG PS**		
0-1	35 (69)	92 (80)
>1	16 (31)	23 (20)
**Immunotherapy**		
Anti-PD1	43 (84)	93 (81)
Anti-PD-L1	8 (16)	15 (13)
Combination**	0 (0)	7 (6)
**Response**		
PD	25 (49)	38 (33)
SD	9 (18)	14 (12)
OR	17 (33)	63 (55)
**Total**	51	115

*ECOG PS: Eastern Cooperative Oncology Group Performance Status; OR: overall response (partial response or complete response); PD: progressive disease; SD: stable disease; TPS: tumor proportion score.

** Combination of Immunotherapy (Durvalumab-Tremelimumab, Pembrolizumab-Feladilimab, Atzeolizumab-Bevacizumab, Atezolizumab-Daratumumab)

Supplementary Table 2. Clinical characteristics of patients according to exhaustion levels. Related to Figure 1

	DESeq2			PFS log-Rank	Risk Score
	**LogFC**	**pvalue**	**padj**	**pvalue**	**Coefficient**
RIMKLB	1.636	5.00E-09	3.95E-05	2.50E-04	-0.101
TMC1	-2.964	1.80E-08	6.98E-05	3.07E-02	0.148
BPIFA1	-2.088	7.09E-07	1.32E-03	2.47E-02	0.032
SCGB1A1	2.701	7.48E-07	1.32E-03	9.49E-05	-0.042
FGA	2.189	5.74E-06	4.96E-03	2.73E-05	0.022
CLDN2	-2.274	2.84E-05	1.41E-02	6.30E-04	0.044
CES5A	2.502	3.92E-05	1.76E-02	6.42E-04	-0.115
GALNT4	1.015	5.02E-05	1.98E-02	1.35E-03	-0.007
AP3B2	-2.091	6.86E-05	2.40E-02	3.26E-02	0.161
GDF15	-1.039	7.27E-05	2.47E-02	3.60E-02	0.168
CLDN20	3.168	1.06E-04	2.87E-02	1.34E-02	-0.151
KCNK5	-1.008	1.00E-04	2.87E-02	4.42E-02	0.051
TFF2	2.087	1.16E-04	3.00E-02	4.22E-03	-0.022
EVA1A	-1.014	2.72E-04	4.85E-02	2.98E-02	0.076
SPZ1	3.207	2.99E-04	4.98E-02	4.19E-03	-0.002
NHLH2	-1.864	3.44E-04	5.33E-02	5.66E-03	0.102
N4BP3	-1.000	3.72E-04	5.50E-02	4.01E-03	0.138
ANKRD22	0.791	3.76E-04	5.52E-02	1.21E-02	-0.431
P2RY13	0.707	4.05E-04	5.66E-02	1.45E-04	-0.052
LRG1	1.036	4.57E-04	6.10E-02	2.78E-02	0.027
PTGDS	0.946	5.03E-04	6.40E-02	3.71E-02	-0.188
TNF	0.968	5.03E-04	6.40E-02	4.78E-02	-0.040
KCNQ3	-1.014	5.24E-04	6.52E-02	2.53E-02	0.001
TIMD4	1.291	7.22E-04	7.85E-02	9.20E-06	-0.147
KIF26B	-0.697	1.01E-03	9.42E-02	4.02E-02	0.113

Supplementary Table 3. CD8+ exhaustion gene signature - 25 genes. Related to Figure 2
