# Supplementary material for: Analysis of PD1, LAG3, TIGIT, and TIM3 expression in human lung adenocarcinoma reveals a 25-gene signature predicting immunotherapy response
Source: Cell Rep Med. 2024 Nov 25;5(12):101831. doi: 10.1016/j.xcrm.2024.101831 (PMC11722093; doi:10.1016/j.xcrm.2024.101831)
Supplement: Document S2. Article plus supplemental information [file mmc2.pdf]

Cell Reports
Medicine

Report

Analysis of PD1, LAG3, TIGIT, and TIM3 expression in human lung adenocarcinoma reveals a 25-gene signature predicting immunotherapy response

Graphical abstract

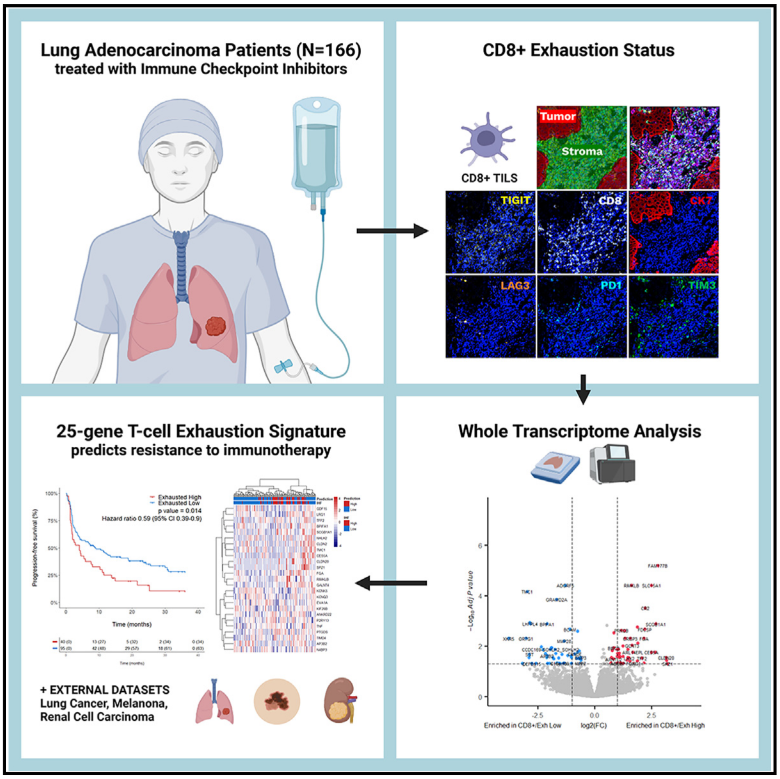

Authors

Jean-Philippe Guégan, Florent Peyraud, Bérengère Dadone-Montaudie, ..., Thomas Grellety, Alban Bessede, Antoine Italiano

Correspondence

a.italiano@bordeaux.unicancer.fr

In brief

In a cohort of 166 patients with lung adenocarcinoma, Guégan et al. show that $CD8^+$ T cell exhaustion is associated with resistance to immune checkpoint inhibitor. They further develop a gene signature that can predict the exhaustion status of $CD8^+$ T cells and the outcome of immunotherapy.

Highlights

- Checkpoint molecules expression on $CD8^+$ cells impacts immunotherapy efficiency
- A 25-gene signature was derived to predict exhausted T cells' abundance
- Exhaustion gene signature predicts outcomes of immunotherapy treatment

Guégan et al., 2024, Cell Reports Medicine 5, 101831
December 17, 2024 © 2024 The Authors. Published by Elsevier Inc.
https://doi.org/10.1016/j.xcrm.2024.101831

Check for updates

CellPress

Cell Reports Medicine

CellPress
OPEN ACCESS

Report

Analysis of PD1, LAG3, TIGIT, and TIM3 expression in human lung adenocarcinoma reveals a 25-gene signature predicting immunotherapy response

Jean-Philippe Guégan,[1] Florent Peyraud,[1,2] Bérengère Dadone-Montaudie,[3] Diego Teyssonneau,[1,2] Lola-Jade Palmieri,[1,2] Emma Clot,[2] Sophie Cousin,[2] Guilhem Roubaud,[2] Mathilde Cabart,[2] Laura Leroy,[2] Coriolan Lebreton,[2] Christophe Rey,[1] Oren Lara,[1] Ophélie Odin,[1] Maxime Brunet,[2] Lucile Vanhersecke,[4] Ezogelin Oflazoglu Gruyters,[5] Ikbel Achour,[5] Leila Belcaid,[6] Sylvestre Le Moulec,[7] Thomas Grellety,[8] Alban Bessede,[1] and Antoine Italiano[2,9,*]

[1]Explicyte Immuno-Oncology, Bordeaux, France
[2]Department of Medicine, Institut Bergonié, Bordeaux, France
[3]Department of Pathology, University Hospital Centre of Nice, Nice, France
[4]Department of Pathology, Institut Bergonié, Bordeaux, France
[5]AstraZeneca, Rahway, NJ, USA
[6]University of Copenhagen, Copenhagen, Denmark
[7]Clinique Marzet, Pau, France
[8]Centre Hospitalier de la Côte Basque, Bayonne, France
[9]Lead contact
*Correspondence: a.italiano@bordeaux.unicancer.fr
https://doi.org/10.1016/j.xcrm.2024.101831

SUMMARY

Immune checkpoint inhibitors (ICIs) have advanced the treatment of non-small cell lung cancer (NSCLC). This study evaluates the predictive value of CD8$^+$ T cell exhaustion in patients with lung adenocarcinoma treated with ICIs. By analyzing tumor samples from 166 patients through multiplex immunofluorescence, we quantify tumor-infiltrating lymphocytes (TILs) expressing exhaustion markers programmed cell death-1 (PD1), lymphocyte activation gene 3 (LAG3), T cell immunoreceptor with Ig and ITIM domains (TIGIT), and T cell immunoglobulin and mucin domain 3 (TIM3). Their co-expression is associated with ICI resistance, irrespective of programmed cell death ligand-1 (PD-L1) status.

We also identify a 25-gene signature indicative of CD8$^+$ T cell exhaustion with high predictive accuracy for ICI response. Validated using several datasets from various clinical trials, this signature accurately predicts ICI responsiveness. Our findings highlight T cell exhaustion's significance in lung adenocarcinoma responses to ICIs and suggest the 25-gene signature as a potential universal biomarker to reinforce precision medicine.

This was registered under Clinical Trial registration number NCT02534649.

INTRODUCTION

The advent of monoclonal antibodies targeting programmed cell death-1 (PD1) or its ligand (PD-L1) has brought about a revolutionary shift in the management of advanced non-small cell lung cancer (NSCLC), with several studies demonstrating notable improvements in objective response rate (ORR), progression-free survival (PFS), and overall survival (OS).[1] However, most patients with NSCLC receiving anti-PD1/PD-L1 monoclonal antibodies do not derive benefit.

PD-L1 expression status as assessed by immunohistochemistry is so far the sole companion diagnostic markers approved both in Europe and in the United States of America to guide anti-PD1 therapy.[2] However, it is an imperfect predictor of a patient's response to immune checkpoint inhibition, as demonstrated by discordant results reported by multiple studies.[2]

The expression of immune checkpoint molecules on T cells, such as PD1, represents a crucial mechanism that the immune system uses to regulate its activity. In addition to PD1, other T cell-associated immune checkpoints have been shown to play an important role in regulating T cell function. Among them, the co-inhibitory receptors lymphocyte activation gene 3 (LAG3), T cell immunoglobulin and mucin domain 3 (TIM3), and T cell immunoreceptor with Ig and ITIM domains (TIGIT) are currently recognized as promising therapeutic targets.[3–5] These receptors are intensively investigated for their clinical activity in combination with PD1/PD-L1 antagonist with the aim to demonstrate a potential synergistic effect and to improve immunotherapy efficacy in patients with advanced NSCLC. As an example, monoclonal antibodies targeting TIGIT have demonstrated a significant improvement in both ORR and PFS when combined with anti-PD1/PD-L1 agents, compared to the use of anti-PD1/PD-L1 as single agents in patients with

This is an open access article under the CC BY license (http://creativecommons.org/licenses/by/4.0/).

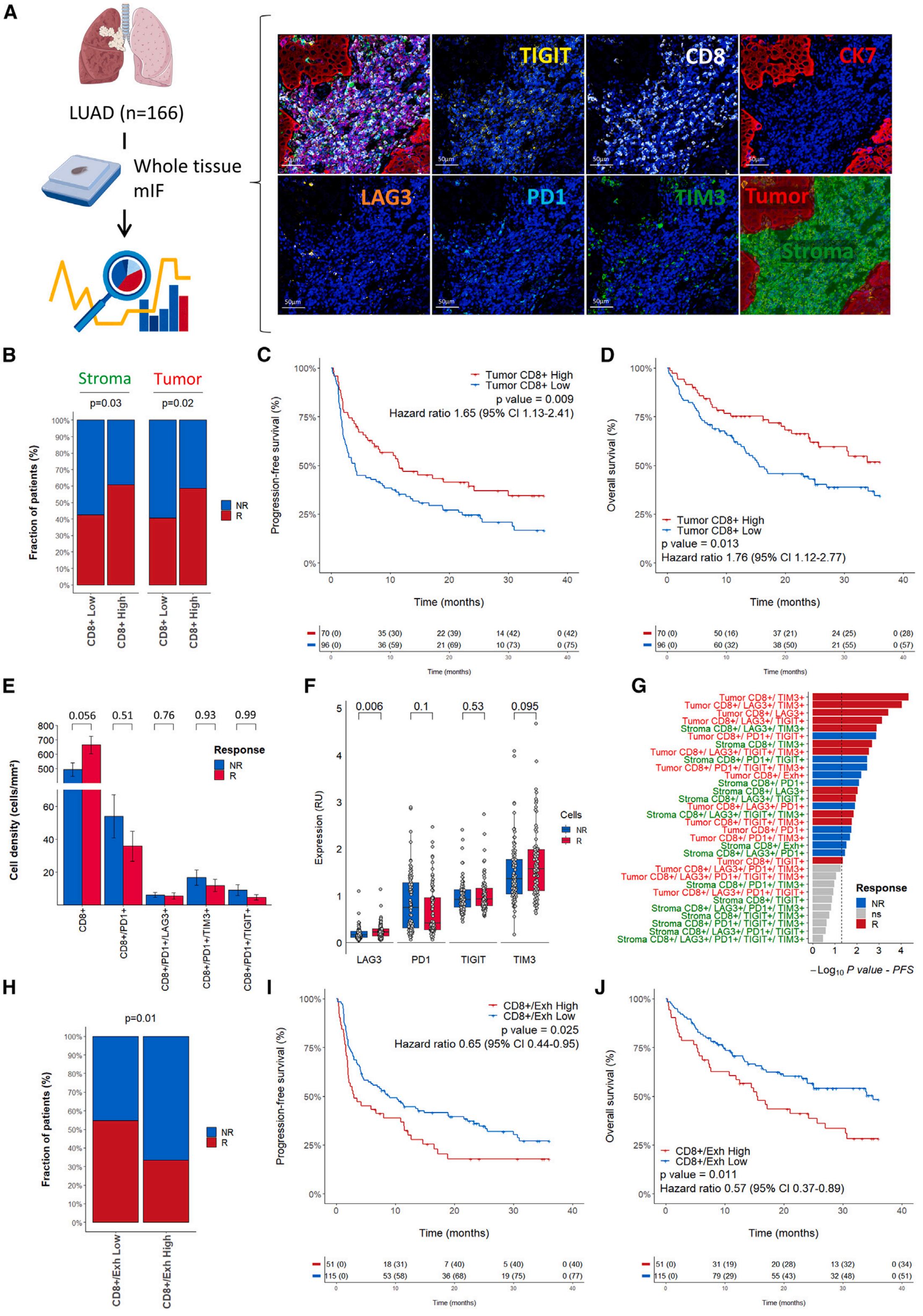

(legend on next page)

chemotherapy-naive, PD-L1-positive, recurrent, or metastatic NSCLC.[6] The influence of $CD8^+$ T cells' immune checkpoint expression profiles on the efficacy of PD1/PD-L1 inhibitors remains uncertain. Additionally, the tumor transcriptomic characteristics associated with the presence of exhausted $CD8^+$ T cells and their relationship to clinical outcomes have yet to be explored. In the context of precision medicine, understanding these dynamics is crucial for advancing immune checkpoint-targeting strategies, including bispecific antibodies (PD1/LAG3, PD1/TIM3, or PD1/TIGIT), now in clinical development. Therefore, we aimed to conduct a comprehensive analysis of the co-expression patterns of PD1, LAG3, TIGIT, and TIM3, their prognostic significance, and their association with tumor transcriptomics in patients with advanced lung adenocarcinoma undergoing treatment with PD1/PD-L1 inhibitors.

RESULTS

Co-expression of exhaustion markers is independently associated with primary resistance to immune checkpoint inhibition

To explore the predictive value of $CD8^+$ T cell exhaustion in patients with lung adenocarcinoma undergoing treatment with immune checkpoint inhibitors (ICIs), we utilized a comprehensive multiplex immunohistofluorescence assay that allows for the assessment of the presence of tumor-infiltrating lymphocytes (TILs) expressing markers indicative of cellular exhaustion (CD8, CK7, LAG3, PD1, TIGIT, and TIM3) on tumor samples obtained before immunotherapy onset from 166 patients with advanced lung adenocarcinoma treated with anti-PD1 or anti-PD-L1 monoclonal antibodies and included prospectively in an institutional tumor profiling program (NCT02534649) (Figure 1A). Response to treatment was assessed based on blinded central review of imaging. The patient characteristics are summarized in Table S1. The median density of $CD8^+$ T cells was 442.5 (6.3–2,341.3) cells/mm^2. As expected, high intra-tumoral CD8 density was significantly associated with improved ORR (58.6 vs. 40.6, p = 0.02), median PFS (11.5 months [95% confidence interval (CI): 7.3–30] vs. 4 months [95% CI: 2.7–9], p = 0.009), and median OS (NA [95% CI: 25.6-NA] vs. 15.6 [95% CI: 13–34], p = 0.013) (Figures 1B–1D); a similar trend for stromal CD8 density was observed (Figure S1). By applying an automated $CD8^+$ single-cell count for immunolabeled PD1, LAG3, TIM3, and TIGIT, we then analyzed the relative abundance of each subset of $CD8^+$ cells and their association with objective response to PD1/PD-L1 inhibition. Patients with high density of $CD8^+$ expressing PD1 alone or together with LAG3, TIM3, or TIGIT tend to have lower response to ICIs, even if statistical significance is not reached (Figure 1E). Similarly, expression of PD1 by $CD8^+$ T cells was increased in non-responder patients compared to responders whereas expression of other co-inhibitory receptors was similar or increased in responders (Figures 1F and S2). Finally, whatever the subset and the compartment considered, patients with primary resistance to treatment were characterized by a highest proportion of $CD8^+$ T cells expressing PD1 and at least one other exhaustion marker immune checkpoint (LAG3 ± TIGIT ± TIM3), herein define as CD8+/Exh (Figure 1G). Patients with tumor highly infiltrated by CD8+/Exh cell (e.g., CD8+/PD1+ ± LAG3+/TIGIT+/TIM3+) had significantly lower response rate (33.3% vs. 54.8%, p = 0.01), PFS (3 months [95% CI: 2–11.5] vs. 9 months [95% CI: 4.6–1], p = 0.025), and OS (15.4 months [95% CI: 10.8–30.5] vs. 35.1 months [95% CI, 23.9-NA], p = 0.011) (Figures 1H–1J).

The comparative analysis of PD-L1 status and genomic alterations between tumors with high versus low abundance of CD8+/Exh showed that PD-L1 expression was comparable in tumors with a high level of exhaustion: 61.5% of these tumors exhibited elevated PD-L1, compared to 57.8% of tumors with low exhaustion. TMB as well as KRAS and other oncogene mutations were also evenly distributed (Figure S3). Similar clinical characteristics were also observed between the two groups (Table S2).

In the multivariate analysis, a high abundance of CD8+/Exh was independently associated with both PFS and OS, with hazard ratios (HRs) of 1.66 (95% CI: 1.13–2.45, p = 0.010) and 1.98 (95% CI: 1.31–3.00, p = 0.001), respectively. This suggests that immune exhaustion may serve as a robust prognostic indicator for poorer outcomes, irrespective of other factors such as age, gender, baseline Eastern Cooperative Oncology Group performance status, prior treatment lines, and PD-L1 status (Table 1).

Figure 1. Exhaustion of $CD8^+$ T cells is predictive of outcome in patients with NSCLC treated with immune checkpoint inhibitors

(A) The presence of tumor-infiltrating lymphocytes expressing exhaustion markers was assessed using a multiplex immunohistofluorescence assay combining CD8, CK7, LAG3, PD1, TIGIT, and TIM3 markers. The images show the expression of each single marker assessed, with its corresponding fluorescence spectrum, and their combination (top left image), respectively.

(B) Histograms of the clinical response achieved in patients with lung adenocarcinoma (LUAD) with « High » versus « Low » density of $CD8^+$ cells in the stroma and tumor areas. Statistical significance was determined by chi-squared test.

(C and D) Kaplan-Meier curves of progression-free survival (C) and overall survival (D) of patients with LUAD treated with ICI according to the level of tumor-infiltrating $CD8^+$ cells. Statistical significance was determined by log rank test.

(E) Histograms of the density of total $CD8^+$ cells expressing exhaustion markers according to clinical response. Statistical significance was determined by Wilcoxon test. Data are represented as mean ± SEM.

(F) Boxplot representation of the relative expression of LAG3, PD1, TIGIT, and TIM3 in $CD8^+$ cells according to clinical response. Statistical significance was determined by Wilcoxon test. Data are represented as median ± interquartiles.

(G) Bar plot of the impact of indicated cell population on PFS of patients with LUAD treated with ICI. Patients were considered as « High » or « Low » based on an optimal cutpoint calculated for each cell population. Statistical significance was determined by log rank test.

(H) Histograms of the clinical response achieved in patients with « High » versus « Low » percentage of exhausted $CD8^+$ cells (CD8+/Exh for CD8+/PD1+ cells expressing LAG3 ± TIGIT ± TIM3). Statistical significance was determined by chi-squared test.

(I and J) Kaplan-Meier curves of progression-free survival (I) and overall survival (J) of patients with LUAD treated with ICI according to the level of exhausted $CD8^+$ cells (CD8+/Exh). Statistical significance was determined by log rank test. NR, non-response; R, response.

To investigate the impact of T cell distribution within the tumor microenvironment on patient outcomes, we performed spatial analysis, measuring distances between "Competent" CD8+ (CD8+/PD1−) and CD8+/Exh (CD8+/PD1+ ± LAG3/TIGIT/TIM3+) cells in relation to tumor (CK7+) cells. The analysis showed that closer proximity of competent CD8+ cells to tumor cells, as well as greater distances between competent CD8+ cells and CD8+/Exh, was significantly linked to better response rates and PFS. In contrast, a closer proximity of CD8+/Exh cells to tumor cells correlated with worse outcomes (Figures S4A–S4I). Furthermore, we explored the correlation between immune phenotype—categorizing tumors into "Desert," "Excluded," and "Infiltrated" phenotypes—and T cell exhaustion status. As expected, the group with low abundance of exhausted CD8+ cells was enriched in "desert" tumors. However, the prevalence of exhausted CD8+ cells did not differ significantly between infiltrated and excluded tumors, suggesting that immune contexture may not be linked to the exhaustion profile of the CD8+ population (Figures S4J–S4L).

A 25-gene signature robustly predicted CD8+ T cell exhaustion levels in patients with lung adenocarcinoma and their response to immunotherapy

To better characterize the processes involved in CD8+ T cell exhaustion, we analyzed the whole transcriptome profile of 135 cases, where exhaustion was measured using multiplexed immunohistofluorescence (mIHF) on formalin-fixed paraffin-embedded (FFPE) serial sections (Figure 2A). This approach enabled the identification of distinct expression patterns associated with CD8+ T cell exhaustion, as demonstrated in the volcano plot (Figure 2B), which revealed differential expression between high and low exhaustion groups, highlighting key genes potentially driving the exhaustion phenotype. Gene Ontology enrichment analysis revealed significant enrichment in inflammation-related biological processes in samples with high exhaustion of CD8+ T cells (Figure 2C). Additionally, single-sample gene set enrichment analysis (ssGSEA) estimation of immune cell populations demonstrated a significant association between CD8+ exhaustion and regulatory T cell infiltration levels within the tumor microenvironment, validated statistically by the Wilcoxon test (Figure 2D). When we compare our bulk RNA sequencing (RNA-seq) dataset with previously published exhaustion T cell signatures, derived from single-cell analyses,[7–15] we found a significant enrichment of these gene signatures with the exhaustion profile measured by IHF. However, each of these signatures, when considered individually, was paradoxically associated with improved patient outcomes in our dataset (Figure 2E). We therefore decided to derive a gene expression signature predictive of T cell exhaustion status and ICI response in patients with lung adenocarcinoma (Figure S5A). By employing a Lasso regression model, we identified a 25-gene exhaustion transcriptomic signature (Figures 2F and Table S3). The optimal model parameters were determined by minimizing the binomial deviance, leading to a precise lambda value that balanced model complexity with prediction accuracy (Figures S5B and S5C). The gene signature's performance in predicting CD8+ exhaustion was highlighted by a receiver operating characteristic (ROC) curve, which yielded an area under the curve of 0.98 in our discovery cohort (Figure 2G) and 0.79 in our validation cohort (Figure S5D), indicating high predictive capability. Kaplan-Meier analysis further demonstrated that patients with a high exhaustion signature had significantly worse PFS compared to those with low exhaustion, emphasizing its potential in predicting resistance to immunotherapy (Figure 2H).

Table 1. Multivariate analysis of predictive factors associated with progression-free survival and overall survival

		PFS		OS	
	n	HR (95% CI)	*p* value	HR (95% CI)	*p* value
Age group					
18–65	85	Ref.	–	Ref.	–
>65	79	1.17 (0.81, 1.68)	0.406	1.11 (0.74, 1.64)	0.620
Gender					
Female	55	Ref.	–	Ref.	–
Male	109	1.14 (0.78, 1.68)	0.496	1.28 (0.83, 1.96)	0.259
Baseline ECOG[a]					
0–1	125	Ref.	–	Ref.	–
2	39	2.74 (1.76, 4.26)	<0.001	5.17 (3.19, 8.40)	<0.001
Prior line					
0	48	Ref.	–	Ref.	–
1	116	1.26 (0.81, 1.95)	0.308	1.04 (0.66, 1.65)	0.865
PD-L1 status					
Negative	64	Ref.	–	Ref.	–
Positive	100	0.42 (0.28, 0.63)	<0.001	0.43 (0.28, 0.67)	<0.001
Exhaustion					
Low	113	Ref.	–	Ref.	–
High	51	1.66 (1.13, 2.45)	0.010	1.98 (1.31, 3.00)	0.001

[a]ECOG PS: Eastern Cooperative Oncology Group Performance Status.

External validation of the 25-gene CD8+ T cell exhaustion signature

To confirm the robustness of our results, we evaluated the predictive value of our signature using two independent datasets: the POPLAR (NCT02517892)/OAK (NCT02008227) randomized trials, which compared immunotherapy with atezolizumab to chemotherapy with docetaxel in patients with advanced NSCLC and the MATCH-R prospective study (NCT02517892) (Figure 2I). In the POPLAR/OAK atezolizumab arm, patients characterized by a low exhaustion signature exhibited significantly better PFS compared to those with a low exhaustion signature, as indicated by a HR of 0.73 and a p value of 0.003. This association was not observed in the chemotherapy arm, where the HR was 1.13 (p value of 0.274), suggesting that the signature's predictive value is specific to immunotherapy responses (Figures 3A and 3B). In the MATCHR dataset, patients

CellPress
OPEN ACCESS

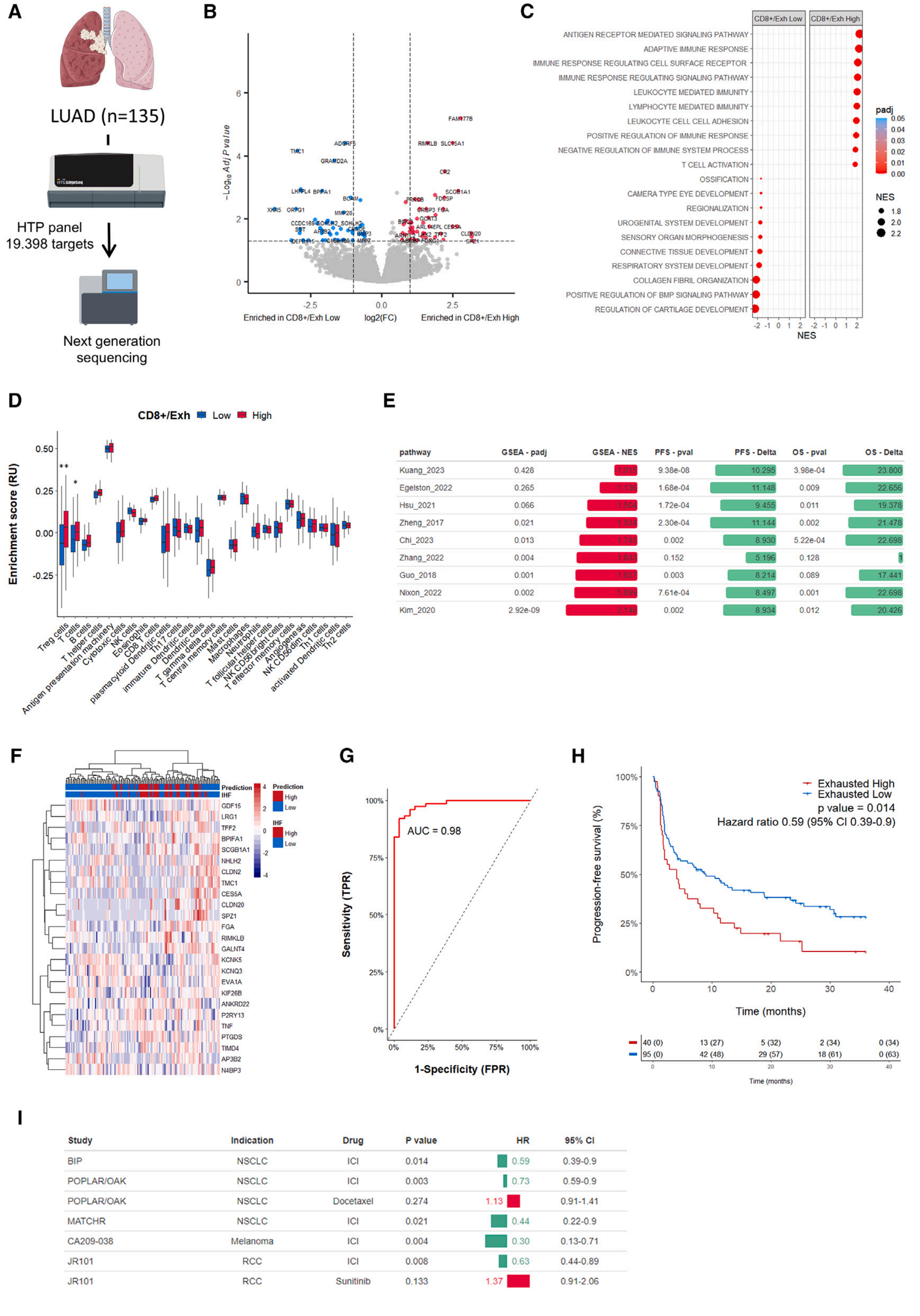

Figure 2. 25-gene signature predicts CD8^{+} exhaustion and resistance to immunotherapy

(A) Transcriptomic profiles of patients with LUAD (N = 135) screened by mIHF for CD8^{+} Exhaustion were assessed using HTG sequencing on an FFPE serial section.

(legend continued on next page)

with advanced NSCLC characterized by a low exhaustion signature exhibited significantly better PFS compared to those with a high exhaustion signature (median PFS: 11.6 months [95% CI: 7-NA] vs. 2 months [95% CI: 1.3–9.6], $p = 0.021$) (Figure 3C). Collectively, these findings confirm the robustness of the CD8$^+$ T cell exhaustion signature as a biomarker for stratifying patients likely to benefit from immunotherapy.

To analyze the impact of CD8$^+$ T cell exhaustion on immunotherapy response, we then calculated the exhaustion score of 15 patients with lung adenocarcinoma for whom tumors were analyzed by RNA-seq at baseline and on-treatment. Interestingly, patients with lower enrichment score tended to experience more durable clinical benefice than patients with a high CD8$^+$ T cell exhaustion score (Figure 4A). Similarly, patients whose exhaustion scores increased during treatment experienced a shorter PFS and a weaker response to immunotherapy (Figures 4B and 4C).

Predictive value of the 25-gene exhaustion signature extends beyond lung adenocarcinoma

We investigated the potential universal applicability of the exhaustion signature across various tumor types. In the CA209-038 trial (NCT01621490), patients with melanoma with a low exhaustion signature experienced significantly longer PFS when treated with nivolumab, evidenced by a HR of 0.30 ($p = 0.004$) (Figure 3D). A similar trend was further echoed in the JAVELIN Renal 101 (NCT0268400601) trial comparing avelumab in combination with axitinib against sunitinib in renal cell carcinoma, where a low exhaustion signature again predicted better PFS (HR 0.63, $p = 0.008$) for patients enrolled in the avelumab-axitinib arm. Interestingly, the signature did not have predictive value in the sunitinib arm, as no statistically significant difference in PFS was observed (HR 1.37, $p = 0.133$) (Figures 3E and 3F).

DISCUSSION

Although various studies have examined the overexpression of different immune checkpoints on T cells and their links to T cell exhaustion in cancers such as colorectal cancer, melanoma, NSCLC, breast cancer, liver cancer, and other tumors, our analysis represents, to the best of our knowledge, the most comprehensive assessment of the co-expression patterns of CD8$^+$ T cell immune checkpoint markers and their impact on immunotherapy efficacy in patients with lung adenocarcinoma.[7–15] We demonstrate that a high abundance of TILs co-expressing PD1 with LAG3, TIGIT, or TIM3 within the tumor microenvironment predicts poorer ORRs, PFS, and OS in patients with NSCLC treated with ICIs, regardless of PD-L1 status.

Our findings align with previous research showing that increased co-expression of PD1 alongside other immune checkpoints leads to diminished antigen sensitivity and a decline in CD8$^+$ TIL functionalities, including reduced cytokine production, proliferation, and cytotoxic capacity. Specifically, the co-expression of TIM3 with PD1 identifies the most severely exhausted subset of CD8$^+$ T cells, which are characterized by their reduced ability to proliferate and secrete IL-2, TNF, and IFN-γ.[16–18] Targeting these inhibitory receptors can rejuvenate T cell activity and enhance anti-cancer effects. Additionally, we observed that the spatial distribution of TILs co-expressing PD1 with LAG3 and/or TIM3 and/or TIGIT influences outcomes, with closer proximity to tumor cells indicating worse clinical benefit. This suggests a targeted, non-random distribution of this TIL subset.

In our study, we identified a transcriptomic signature distinguishing lung adenocarcinoma with high vs. low abundance of exhausted T cells, utilizing bulk transcriptomic analysis to provide an overview of the transcriptional landscape in lung adenocarcinomas with varying levels of T cell exhaustion. Interestingly, CD8$^+$ T cell exhaustion signatures previously reported from single-cell analyses,[7–15] though enriched in our transcriptomic signature, were paradoxically associated with improved outcomes on immunotherapy in our dataset. This paradox underscores the notion that these signatures represent not only immune dysfunction but also an active immune engagement with the tumor, a prerequisite for the efficacy of anti-PD1 therapies.

The bulk transcriptomic analysis approach we employed bridges experimental and clinical realms. While single-cell RNA sequencing offers detailed mechanistic insights for identifying biomarkers and therapeutic targets, bulk RNA-seq provides a scalable and cost-effective alternative for clinical diagnostics. Our signature suggests that T cell exhaustion in lung adenocarcinomas stems from a complex network of biological processes, highlighting potential therapeutic intervention targets. For example, the inclusion of the *TNF* gene in our signature points to a state of heightened inflammation within NSCLC tumors, contributing to T cell exhaustion through sustained upregulation of inhibitory checkpoints.[19] This reflects the dynamic interplay between pro- and anti-tumorigenic forces within the immune landscape of lung adenocarcinoma.

(B) Volcano plot of the genes differentially expressed between CD8$^+$ exhaustion "High" and "Low" patients.
(C) Bubble plot of Gene Ontology terms enrichment in patients with "High" or "Low" CD8$^+$ exhaustion.
(D) Immune cell estimation by ssGSEA according to the level of CD8$^+$ exhaustion. Statistical significance was determined by Wilcoxon test (** ≤ 0.01, * ≤ 0.05). Data are represented as median ± interquartiles.
(E) GSEA of exhaustion genes signatures and impact on PFS and OS of patients with LUAD treated with ICI. Statistical significance of PFS and OS was determined by log rank tests. Patients were classified as "High" or "Low" based on optimal cutpoint determined for each exhaustion score.
(F) Heatmap of the genes included in the exhaustion signature. Gene signature-based prediction of CD8$^+$ exhaustion and levels assessed by mIHF are annotated for each patient.
(G) ROC curve of the CD8$^+$ exhaustion prediction in the discovery cohort ($N = 100$ patients).
(H) Kaplan-Meier curves of progression-free survival of patients with LUAD ($N = 135$) treated with ICI according to predicted level of CD8$^+$ exhaustion. Statistical significance was determined by log rank test.
(I) Impact of 25-gene signature score on PFS of patients with cancer according to treatment. Statistical significance of PFS was determined by log rank test. Patients were classified as "High" or "Low" based on optimal cutpoint determined for each study.

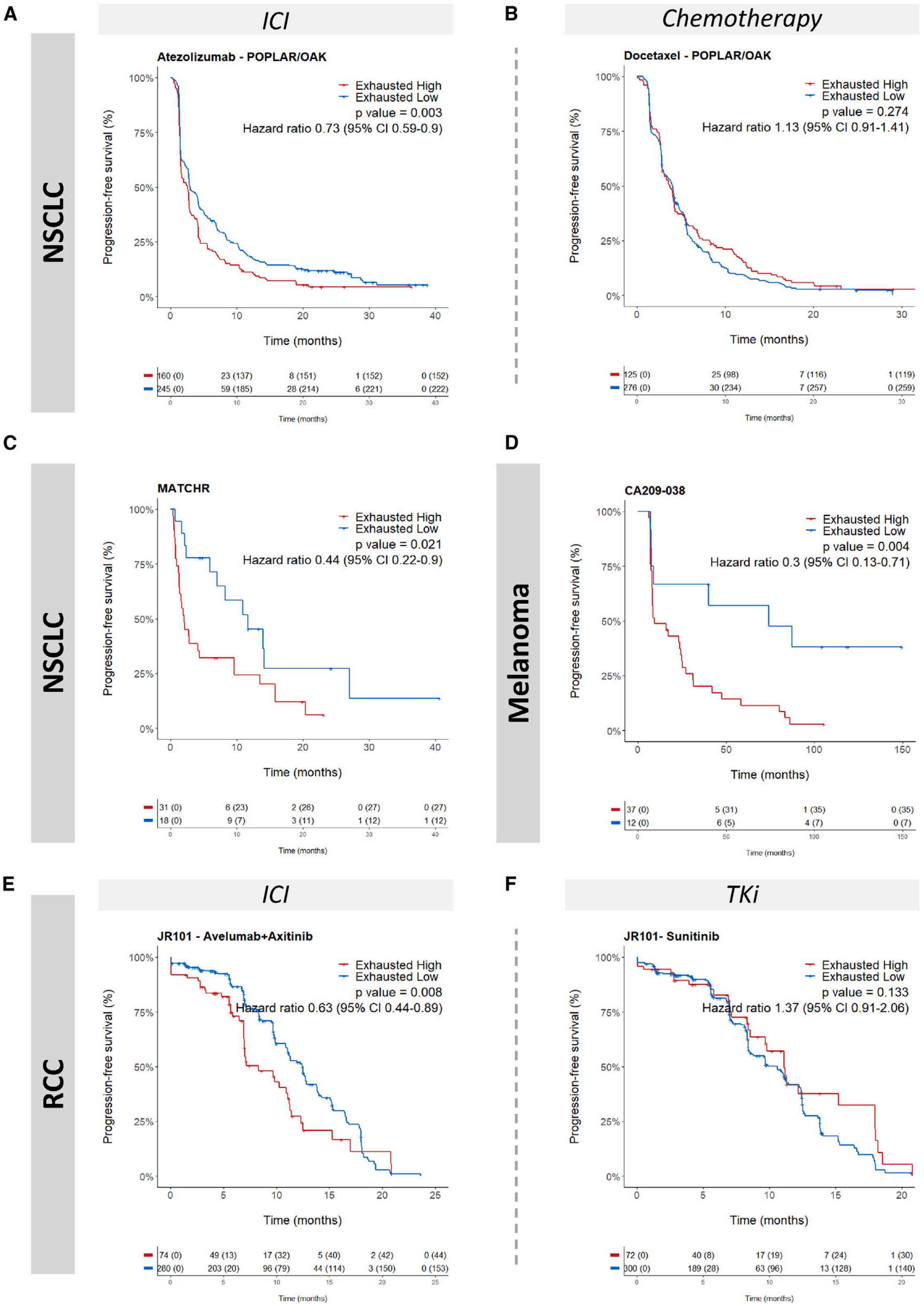

(legend on next page)

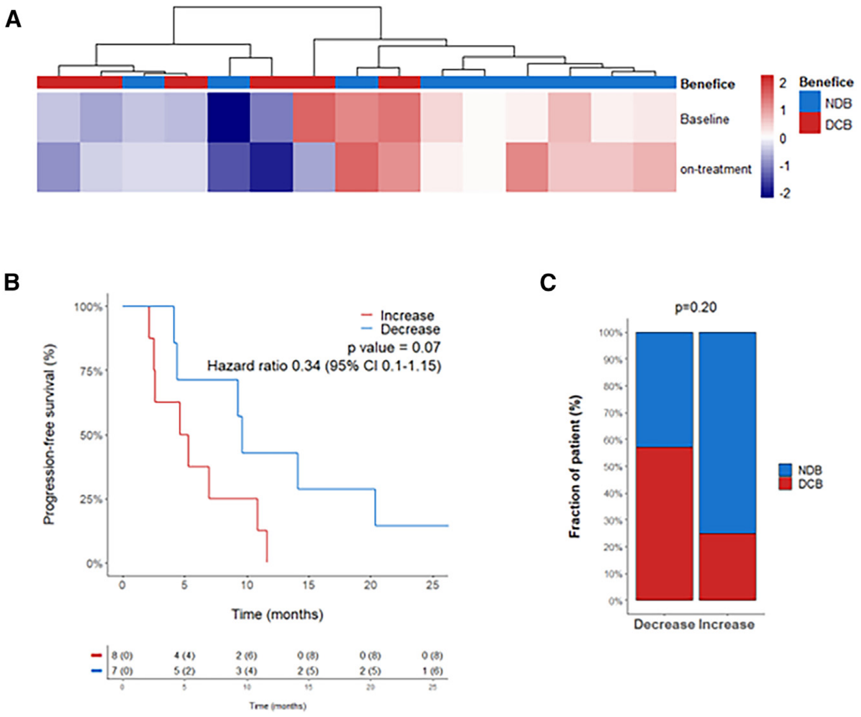

Figure 4. Enrichment of exhaustion signature on-treatment is associated with poorer prognosis

Tumors of 15 patients with NSCLC treated with immunotherapy were collected at baseline or on-treatment and analyzed by RNA-seq.

(A) Heatmap visualization of enrichment scores of exhaustion signature.

(B) Kaplan-Meier curve of progression-free survival of patients classified as "Increase" or "Decrease" according to the ratio of exhaustion signature score between baseline and on-treatment. Statistical significance was determined by log rank test.

(C) Histograms of the clinical benefit achieved in patients with "Increase" versus "Decrease" exhaustion signature over time. Statistical significance was determined by chi-squared test. NDB: "non-durable clinical benefit"; DCB, "durable clinical benefit."

Moreover, the presence of genes such as *CLDN2* and *GALNT4* in our signature reveals an intricate network where adhesion molecules, metabolic enzymes, and immune regulatory factors converge (Figure S5E), indicating a systemic reprogramming of the immune response.[20,21] High expression of our signature independently correlated with outcomes in patients with NSCLC receiving immunotherapy, underscoring its specificity in predicting responses to immunotherapeutic interventions rather than to chemotherapy. This pattern was consistent in patients with advanced renal cancer, where the signature predicted immunotherapy effectiveness but did not predict responses to tyrosine kinase inhibitor treatments. The confirmation of our signature's predictive value across independent datasets and a range of tumor types emphasizes the broad applicability and significance of T cell exhaustion as a biomarker for immunotherapy outcomes.

Crucially, our findings challenge the traditional view that immune exhaustion solely predicts poor outcomes. Indeed, our signature, as a surrogate of the exhausted T cell phenotype, offers a therapeutic opportunity. Bispecific antibodies that simultaneously target PD1 and other inhibitory receptors like TIGIT, TIM3, or LAG3 are designed to reinvigorate these exhausted T cells, potentially restoring their anti-tumor functionality. Our study thus provides a molecularly informed basis for patient stratification for this new class of agents, underscoring the need for further investigation of our 25-gene expression signature in the context of ongoing clinical development of bispecific antibodies targeting PD1 and other immune checkpoints.

Limitations of the study

One key limitation of our study is its retrospective nature, which may limit the immediate clinical applicability of our findings. Although we validated our 25-gene exhaustion signature using independent datasets, prospective validation in future clinical trials is necessary to confirm its predictive value. Without such validation, the generalizability of the signature across broader patient populations and varying treatment regimens remains uncertain. Conducting prospective studies will be critical in establishing the clinical utility of this biomarker for guiding immunotherapy decisions.

RESOURCE AVAILABILITY

Lead contact

Further information and requests for resources and reagents should be directed to the lead contact Antoine Italiano (a.italiano@bordeaux.unicancer.fr).

Materials availability

All reagents/materials generated in this study will be made available upon request. The request may require a completed Material Transfer Agreement.

Figure 3. Exhaustion signature predicts response to immunotherapy only across different tumor types

(A–F) Kaplan-Meier curves of progression-free survival of patients with NSCLC (A–C), melanoma (D), or renal cell carcinoma (RCC) (E and F) treated with ICI (A, C, D, and E), chemotherapy (B), or TKi (F). Patients were classified as "High" or "Low" based on optimal cutpoint. Statistical significance was determined by log rank test.

Data and code availability

- The datasets used in this study are from a clinical trial conducted at Institut Bergonié, Bordeaux, France, and are not publicly available due to potential privacy concerns related to research participants. Access to these datasets is governed by French/European regulations, and any reuse of the data must be approved by the ethics committee "CPP du Sud-Ouest et d'Outre-Mer III," Bordeaux, France. To request access to the data, please contact the lead contact (a.italiano@bordeaux.unicancer.fr). Proposals for data access may be submitted up to 36 months following the publication of this article. Requests must include a detailed description of the research objectives and will be considered for non-commercial, research purposes only. In order to protect participant privacy, no personally identifiable or sensitive clinical information will be provided. All requests must comply with the consent agreements established with study participants. This paper does not report original code.
- Any additional information required to reanalyze the data reported in this paper is available from the lead contact upon request.

ACKNOWLEDGMENTS

This study was funded by AstraZeneca and Conseil Régional Aquitaine.

AUTHOR CONTRIBUTIONS

Conceptualization, A.I., A.B., and J.-P.G.; methodology, A.I., A.B., and J.-P.G.; investigations, F.P., B.D.-M., D.T., L.-J.P., E.C., S.C., G.R., M.C., L.L., C.L., C.R., O.L., O.O., M.B., L.V., L.B., S.L.M., and T.G.; writing, A.I., A.B., J.-P.G., and F.P.; funding acquisition, E.O.G. and I.A.; supervision, A.I. and A.B.

DECLARATION OF INTERESTS

A.B., J.-P.G., C.R., O.L., and O.O. are employees of ImmuSmol/Explicyte. E.O.G. and I.A. are employees of AstraZeneca. A.I. received research grants from AstraZeneca, Bayer, BMS, Chugai, Merck, MSD, PharmaMar, Novartis, and Roche, and received personal fees from Epizyme, Bayer, Lilly, Roche, and Springworks.

STAR★METHODS

Detailed methods are provided in the online version of this paper and include the following:

- KEY RESOURCES TABLE
- EXPERIMENTAL MODEL AND STUDY PARTICIPANT DETAILS
  - Patient
- METHOD DETAILS
  - Treatments and evaluation
  - Immunohistofluorescence
  - PD-L1 scoring
  - Mutational status
  - Transcriptomic analyses
  - RNAseq data access
- QUANTIFICATION AND STATISTICAL ANALYSIS

SUPPLEMENTAL INFORMATION

Supplemental information can be found online at https://doi.org/10.1016/j.xcrm.2024.101831.

Received: April 18, 2024
Revised: June 3, 2024
Accepted: October 28, 2024
Published: November 25, 2024

REFERENCES

1. Reck, M., Remon, J., and Hellmann, M.D. (2022). First-Line Immunotherapy for Non-Small-Cell Lung Cancer. J. Clin. Oncol. *40*, 586–597. https://doi.org/10.1200/JCO.21.01497.
2. Davis, A.A., and Patel, V.G. (2019). The role of PD-L1 expression as a predictive biomarker: an analysis of all US Food and Drug Administration (FDA) approvals of immune checkpoint inhibitors. J. Immunother. Cancer *7*, 278.
3. Das, M., Zhu, C., and Kuchroo, V.K. (2017). TIM3 and its Role in Regulating Anti-Tumor Immunity. Immunol. Rev. *276*, 97–111.
4. Andrews, L.P., Marciscano, A.E., Drake, C.G., and Vignali, D.A.A. (2017). LAG3 (CD223) as a Cancer Immunotherapy Target. Immunol. Rev. *276*, 80–96.
5. Chauvin, J.-M., and Zarour, H.M. (2020). TIGIT in Cancer Immunotherapy. J. Immunother. Cancer *8*, e000957.
6. Johnson, M.L., Fox, W., Lee, Y.G., Lee, K.H., Ahn, H.K., Kim, Y.-C., Lee, K.-Y., Lee, J.-S., He, X., Park, C., et al. (2022). ARC-7: Randomized phase 2 study of domvanalimab + zimberelimab ± etrumadenant vs zimberelimab in first-line, metastatic, PD-L1–high non–small cell lung cancer. ASCO Plenary Series *40*. https://doi.org/10.1200/JCO.2022.40.36_suppl.39760.
7. Kuang, T., Zhang, L., Chai, D., Chen, C., and Wang, W. (2023). Construction of a T-cell exhaustion-related gene signature for predicting prognosis and immune response in hepatocellular carcinoma. Aging (Albany NY) *15*, 5751–5774.
8. Egelston, C.A., Guo, W., Tan, J., Avalos, C., Simons, D.L., Lim, M.H., Huang, Y.J., Nelson, M.S., Chowdhury, A., Schmolze, D.B., et al. (2022). Tumor-infiltrating exhausted CD8+ T cells dictate reduced survival in premenopausal estrogen receptor-positive breast cancer. JCI Insight *7*, e153963.
9. Hsu, C.L., Ou, D.L., Bai, L.Y., Chen, C.W., Lin, L., Huang, S.F., Cheng, A.L., Jeng, Y.M., and Hsu, C. (2021). Exploring Markers of Exhausted CD8 T Cells to Predict Response to Immune Checkpoint Inhibitor Therapy for Hepatocellular Carcinoma. Liver Cancer *10*, 346–359.
10. Zheng, C., Zheng, L., Yoo, J.K., Guo, H., Zhang, Y., Guo, X., Kang, B., Hu, R., Huang, J.Y., Zhang, Q., et al. (2017). Landscape of Infiltrating T Cells in Liver Cancer Revealed by Single-Cell Sequencing. Cell *169*, 1342–1356.e16.
11. Chi, H., Zhao, S., Yang, J., Gao, X., Peng, G., Zhang, J., Xie, X., Song, G., Xu, K., Xia, Z., et al. (2023). T-cell exhaustion signatures characterize the immune landscape and predict HCC prognosis *via* integrating single-cell RNA-seq and bulk RNA-sequencing. Front. Immunol. *14*, 1137025.
12. Zhang, Z., Chen, L., Chen, H., Zhao, J., Li, K., Sun, J., and Zhou, M. (2022). Pan-cancer landscape of T-cell exhaustion heterogeneity within the tumor microenvironment revealed a progressive roadmap of hierarchical dysfunction associated with prognosis and therapeutic efficacy. EBioMedicine *83*, 104207.
13. Nixon, B.G., Kuo, F., Ji, L., Liu, M., Capistrano, K., Do, M., Franklin, R.A., Wu, X., Kansler, E.R., Srivastava, R.M., et al. (2022). Tumor-associated macrophages expressing the transcription factor IRF8 promote T cell exhaustion in cancer. Immunity *55*, 2044–2058.e5.
14. Guo, X., Zhang, Y., Zheng, L., Zheng, C., Song, J., Zhang, Q., Kang, B., Liu, Z., Jin, L., Xing, R., et al. (2018). Global characterization of T cells in non-small-cell lung cancer by single-cell sequencing. Nat. Med. *24*, 978–985.
15. Kim, K., Park, S., Park, S.Y., Kim, G., Park, S.M., Cho, J.W., Kim, D.H., Park, Y.M., Koh, Y.W., Kim, H.R., et al. (2020). Single-cell transcriptome analysis reveals TOX as a promoting factor for T cell exhaustion and a predictor for anti-PD-1 responses in human cancer. Genome Med. *12*, 22.
16. Andrews, L.P., Yano, H., and Vignali, D.A.A. (2019). Inhibitory receptors and ligands beyond PD-1, PD-L1 and CTLA-4: breakthroughs or backups. Nat. Immunol. *20*, 1425–1434.

17. Jin, H.T., Anderson, A.C., Tan, W.G., West, E.E., Ha, S.J., Araki, K., Freeman, G.J., Kuchroo, V.K., and Ahmed, R. (2010). Cooperation of Tim-3 and PD-1 in CD8 T-cell exhaustion during chronic viral infection. Proc. Natl. Acad. Sci. USA *107*, 14733–14738.
18. Joller, N., Anderson, A.C., and Kuchroo, V.K. (2024). LAG-3, TIM-3, and TIGIT: Distinct functions in immune regulation. Immunity *57*, 206–222.
19. Chen, A.Y., Wolchok, J.D., and Bass, A.R. (2021). TNF in the era of immune checkpoint inhibitors: friend or foe? Nat. Rev. Rheumatol. *17*, 213–223.
20. Ahmad, R., Chaturvedi, R., Olivares-Villagómez, D., Habib, T., Asim, M., Shivesh, P., Polk, D.B., Wilson, K.T., Washington, M.K., Van Kaer, L., et al. (2014). Targeted colonic claudin-2 expression renders resistance to epithelial injury, induces immune suppression, and protects from colitis. Mucosal Immunol. *7*, 1340–1353.
21. Cai, L., Li, Y., Tan, J., Xu, L., and Li, Y. (2023). Targeting LAG-3, TIM-3, and TIGIT for cancer immunotherapy. J. Hematol. Oncol. *16*, 101.
22. Riaz, N., Havel, J.J., Makarov, V., Desrichard, A., Urba, W.J., Sims, J.S., Hodi, F.S., Martín-Algarra, S., Mandal, R., Sharfman, W.H., et al. (2017). Tumor and Microenvironment Evolution during Immunotherapy with Nivolumab. Cell *171*, 934–949.e16.
23. Motzer, R.J., Robbins, P.B., Powles, T., Albiges, L., Haanen, J.B., Larkin, J., Mu, X.J., Ching, K.A., Uemura, M., Pal, S.K., et al. (2020). Avelumab plus axitinib versus sunitinib in advanced renal cell carcinoma: biomarker analysis of the phase 3 JAVELIN Renal 101 trial. Nat. Med. *26*, 1733–1741.
24. Loriot, Y., Marabelle, A., Guégan, J.P., Danlos, F.X., Besse, B., Chaput, N., Massard, C., Planchard, D., Robert, C., Even, C., et al. (2021). Plasma proteomics identifies leukemia inhibitory factor (LIF) as a novel predictive biomarker of immune-checkpoint blockade resistance. Ann. Oncol. *32*, 1381–1390.
25. Bindea, G., Mlecnik, B., Tosolini, M., Kirilovsky, A., Waldner, M., Obenauf, A.C., Angell, H., Fredriksen, T., Lafontaine, L., Berger, A., et al. (2013). Spatiotemporal Dynamics of Intratumoral Immune Cells Reveal the Immune Landscape in Human Cancer. Immunity *39*, 782–795.
26. Pomaznoy, M., Ha, B., and Peters, B. (2018). GOnet: a tool for interactive Gene Ontology analysis. BMC Bioinf. *19*, 470.
27. Eisenhauer, E.A., Therasse, P., Bogaerts, J., Schwartz, L.H., Sargent, D., Ford, R., Dancey, J., Arbuck, S., Gwyther, S., Mooney, M., et al. (2009). New response evaluation criteria in solid tumors: revised RECIST guideline (version 1.1). Eur. J. Cancer *45*, 228–247.
28. Rimm, D.L., Han, G., Taube, J.M., Yi, E.S., Bridge, J.A., Flieder, D.B., Homer, R., West, W.W., Wu, H., Roden, A.C., et al. (2017). A Prospective, Multi-institutional, Pathologist-Based Assessment of 4 Immunohistochemistry Assays for PD-L1 Expression in Non-Small Cell Lung Cancer. JAMA Oncol. *3*, 1051–1058.
29. Milbury, C.A., Creeden, J., Yip, W.K., Smith, D.L., Pattani, V., Maxwell, K., Sawchyn, B., Gjoerup, O., Meng, W., Skoletsky, J., et al. (2022). Clinical and analytical validation of FoundationOne®CDx, a comprehensive genomic profiling assay for solid tumors. PLoS One *17*, e0264138.
30. Bessede, A., Peyraud, F., Besse, B., Cousin, S., Cabart, M., Chomy, F., Rey, C., Lara, O., Odin, O., Nafia, I., et al. (2024). TROP2 Is Associated with Primary Resistance to Immune Checkpoint Inhibition in Patients with Advanced Non-Small Cell Lung Cancer. Clin. Cancer Res. *30*, 779–785.

CellPress
OPEN ACCESS

STAR★METHODS

KEY RESOURCES TABLE

REAGENT or RESOURCE	SOURCE	IDENTIFIER
Antibodies		
anti-PD-1 – Clone NAT105	Roche	07099029001
CONFIRM anti-Cytokeratin 7 – Clone SP52	Roche	05986818001
anti-TIM-3 – Clone D5D5R^{TM}	Cell Signaling Technology	45208
anti-LAG-3 – Clone EP294	BioSB	BSB 3366
anti-CD8 – Clone C8/144B	Agilent Dako	GA62361-2
anti-TIGIT – Clone BLR047F	Abcam	RRID:AB_2943164
DISCOVERY OmniMap anti-Rb HRP	Roche	RRID:AB_2811043
DISCOVERY OmniMap anti-Ms HRP	Roche	RRID:AB_2885182
anti-PD-L1 – Clone QR1	Diagomics	C-P001-01
Chemicals, peptides, and recombinant proteins		
Opal 6-Plex Manual Detection Kit	Akoya Bioscience	SKU NEL861001KT
DISCOVERY ChromoMap DAB Kit	Roche	05266645001
Critical commercial assays		
HTG Transcriptome Panel	HTG Molecular Diagnostics	HTG-001-024
Deposited data		
CA209-038 RNAseq data	Riaz et al.[22]	https://github.com/riazn/bms038_analysis/
JAVELIN Renal 101 RNAseq data	Motzer et al.[23]	https://doi.org/10.1038/s41591-020-1044-8
POPLAR/OAK RNAseq data	European Genome-phenome Archive	EGA: EGAS00001005013
MATCHR RNAseq data	Loriot et al.[24]	https://doi.org/10.1016/j.annonc.2021.08.1748
Immune cell estimation signature	Bindea et al.[25]	https://doi.org/10.1016/j.immuni.2013.10.003
Exhaustion gene signature	Kuang et al.[7]	https://doi.org/10.18632/aging.204830
Exhaustion gene signature	Egelston et al.[8]	https://doi.org/10.1172/jci.insight.153963
Exhaustion gene signature	Hsu et al.,[9]	https://doi.org/10.1159/000515305
Exhaustion gene signature	Zheng et al.[10]	https://doi.org/10.1016/j.cell.2017.05.035
Exhaustion gene signature	Chi et al.[11]	https://doi.org/10.3389/fimmu.2023.1137025
Exhaustion gene signature	Zhang et al.[12]	https://doi.org/10.1016/j.ebiom.2022.104207
Exhaustion gene signature	Guo et al.[14]	https://doi.org/10.1038/s41591-018-0045-3
Exhaustion gene signature	Nixon et al.[13]	https://doi.org/10.1016/j.immuni.2022.10.002
Exhaustion gene signature	Kim et al.[15]	https://doi.org/10.1038/s41467-020-16164-1
Software and algorithms		
inForm Advanced Image Analysis (v2.4.6)	Akoya Bioscience	https://www.akoyabio.com/support/software/
PhenoChart (v1.1.0)	Akoya Bioscience	https://www.akoyabio.com/support/software/
FlowJo (v10.8.0)	FlowJo	https://www.flowjo.com/
R (v4.3.2)	The R Foundation	https://www.r-project.org/
GOnet tool	Pomaznoy et al.[26]	https://tools.dice-database.org/GOnet/
HTG EdgeSeq Reveal Software (v5.4.0.7543)	HTG Molecular Diagnostics	https://www.htgmolecular.com/

EXPERIMENTAL MODEL AND STUDY PARTICIPANT DETAILS

Patient

This study was based on the analysis of data from one prospective precision medicine study (BIP study, Sponsor: Institut Bergonié, Bordeaux, France, NCT02534649). The study was approved by the Comité de Protection des Personnes (CPP) Sud-Ouest et Outre

Mer III. Patients characteristics are described in Table S1. All patients were treated as per standard of care between December 2013 and June 2022. The inclusion criteria were age ≥18 years, histologically proven lung adenocarcinoma, unresectable and/or metastatic disease, no oncogenic addiction, at least one tumor evaluation by imaging after immunotherapy onset, and availability of paraffin-embedded tumor material obtained before immunotherapy onset. Institutional ethics review board approval and patient informed consent were obtained for this study.

METHOD DETAILS

Treatments and evaluation

Patients were treated at the discretion of their physician. The best response to treatment was evaluated according to Response Evaluation Criteria in Solid Tumors (RECIST) after central review.[27] Routine follow-up was similar across the two cohorts included in the study. PFS was defined as the time from the start of treatment until disease progression, death, or last patient contact. OS was defined as the time from the start of treatment until death or last patient contact.

Immunohistofluorescence

To setup multiplexed immunohistofluorescence (mIHF) panel, the staining condition of each antibody was first optimized by immunohistochemistry and the staining pattern was validated by a pathologist. Position of each antibody in the mIF panel was then chosen after characterization of the antibody's resistance for several stripping cycles. Combination of each antibody with specific Opal fluorochrome was performed according to the level of target expression and antibody position in the panel, in order to limit signal spillover. Validation of mIHF panel was finally achieved through the comparison of the signals obtained from monoplex staining with mIHF staining.

Multiplex immunohistofluorescence was performed on whole tissue sections using the following antibodies: PD1 (NAT105, Cell Marque, ready-to-use), CK7 (SP52, Ventana, ready-to-use), TIM3 (D5D5R, Cell Signaling Technology, 1/100), LAG3 (EP294, BioSB, 1/100), CD8 (C8/144B, Dako, 1/25), TIGIT (BLR047F, Abcam, 1/300). Bound primary antibodies were detected using OmniMap anti-Rb HRP (760–4311, Ventana) and OmniMap anti-Ms HRP (760–4310, Ventana) detection kits followed by TSA opal fluorophores (Opal 480, Opal 520, Opal 570, Opal 620, Opal 690 and Opal 780, Akoya Bioscience) according to manufacturer's instructions. The slides were counterstained with spectral DAPI (Akoya Bioscience) and cover-slipped. The slides were digitalized using the PhenoImager HT System (Akoya), and the multispectral images obtained were unmixed using spectral libraries that were previously built from images stained for each fluorophore (monoplex), using the inForm Advanced Image Analysis software (inForm 2.4.6, Akoya Bioscience).

Tumor tissue areas were delineated in PhenoChart (Akoya Bioscience) and analyzed using Inform software to segment the tissue in "Tumor" and "Stroma" areas, based mainly on CK7 staining, and extract for each cell the mean marker intensity. To limit the bias, marker intensity normalization was performed using the "GaussNorm" function from the flowstat R package (v4.8.2) and cells were phenotyped using a thresholding approach in FlowJo (v10.8.0). Distances between CK7+ and $CD8^+$ cells were computed using the phenoptr R package (v0.3.2).

PD-L1 scoring

PDL1 staining was performed by immunohistochemistry on the Ventana Discovery platform using the QR1 clone (Diagomics, 1/100) with the RUO discovery protocol according to the manufacturer's recommendations. Primary antibody was revealed using the detection kits OmniMap anti-Rb HRP (Ventana) followed by DAB chromogenic detection kit (Ventana) according to manufacturer's instructions. The slides were finally counterstained with hematoxylin and digitalized using the PhenoImager HT System (Akoya). The PD-L1 status was determined with the TPS (tumor positive score) following the guidelines.[28] Only viable tumor cells displaying partial or complete staining for PD-L1 membrane expression were considered relative to the total number of tumor cells.

Mutational status

Mutational status assessment used the FDA approved FoundationOne CDx on DNA from FFPE samples, with sequencing on the Illumina NovaSeq 6000 platform, targeting all coding exons of 309 cancer-related genes, detecting a total of 324 gene alterations.[29]

Transcriptomic analyses

Transcriptomic analysis on FFPE samples utilized the HTG Transcriptome Panel (HTP) for a quantitative nuclease protection assay according to HTG Molecular Diagnostics' protocol. The HTP features 19,616 Nucleic Acid Protection Probes (NPPs), including 19,398 target probes, 100 negative controls, 92 RNA controls established by the ERCC, 22 gDNA probes, and 4 positive control (POS) probes. Tissue sections from 5μm FFPE slides were subjected to macrodissection, lysis, Proteinase K treatment for 20 min at 56°C, followed by DNase digestion at 37°C for 30 min. The quantitative nuclease protection assay was processed using HTG EdgeSeq Processors, with PCR amplification adding adapters and sample tags. Sequencing was performed on an Illumina NextSeq 2000 (Flow cell P2 - 100 cycles), and the resulting FASTQ files were processed to a gene expression count matrix using HTG EdgeSeq Reveal Software.

Immune cell estimates were conducted via the ssGSEA framework with the consensusTME R package (v0.0.1.9), utilizing the Bindea dataset.[25] Gene Ontology term and exhaustion signature enrichment analyses were performed using the fgsea R package (v1.28.0), with volcano plot and heatmap visualizations generated by EnhancedVolcano (v1.20.0) and pheatmap (v1.0.12), respectively.

To develop a gene signature that predicts $CD8^+$ exhaustion, we analyzed genes differentially expressed (DEGs) between CD8+/Exhausted High and Low patients using the DESeq2 R package (v1.42.0). We established Benjamini & Hochberg (BH) adjusted *p*-values $\leq$0.10 and |log fold change (FC)| $\geq$0.58 as criteria for selecting DEGs. The prognostic impact of each DEG on immunotherapy response was assessed through univariate analysis using a Cox regression model for progression-free survival. DEGs with a log rank *p*-value $\leq$0.05 were included in a LASSO regression model implemented via the glmnet R package (v4.1-8). The study population was divided into discovery ($n = 100$ patients) and validation ($n = 35$ patients) cohorts. This division was made to ensure an independent test set that could provide an unbiased evaluation of the predictive performance of the 25-gene $CD8^+$ T cell exhaustion signature.

The gene signature was rigorously evaluated through 10-fold cross-validation within the discovery cohort to optimize the model and prevent overfitting. This method involves partitioning the discovery cohort into ten subsets, using nine for training and one for testing, iteratively, which helps in assessing the stability and reliability of the gene signature.

Following the refinement and confirmation of the signature within the discovery cohort, it was subsequently applied to the validation cohort of 35 patients. This step was critical to confirming the signature's predictive capability in an entirely separate group of patients, enhancing the generalizability of our findings. Multifactorial regression coefficients for each gene were used to construct a risk score equation. Risk scores were calculated with the formula Risk $= \sum_i^n \text{Exp}\, i * \beta i$, where Exp*i* denotes the log2(x+1) DESeq2 normalized expression of each gene, and β*i* is the coefficient of each gene. ROC curves for the model were plotted for both discovery and validation cohorts to assess the model's sensitivity and specificity. The association of gene signature modules with Gene Ontology terms was determined using the GOnet tool[26]

RNAseq data access

The CA209-038 study data, including gene expression and clinical data, were downloaded from the publication by Riaz et al.[22] available at Riaz et al. GitHub Repository. Clinical and RNA-seq data from the JAVELIN Renal 101 trial were obtained from the publication by Motzer et al.[23] The POPLAR (GO28753) and OAK (GO28915) clinical and RNAseq data were accessed from the European Genome-Phenome Archive (EGAC00001002120) and processed as we described previously.[30] The MATCHR (NCT02517892) dataset was prepared following the methods outlined in Loriot et al.[24]

QUANTIFICATION AND STATISTICAL ANALYSIS

The cutoff date for the statistical analysis of baseline demographic data and clinical outcomes was 09/30/2022. Survival rates were estimated using the Kaplan–Meier method. Patients were classified as "High" or "Low" based on an optimized threshold obtained using the maximally selected rank statistics from the maxstat R package, with progression-free survival (PFS) as the optimal outcome (survminer R package v0.4.9). Survival differences between groups were assessed using the log rank test (survival R package v3.3.1). The Cox proportional-hazards regression model was used to estimate hazard ratios (HR) and 95% confidence intervals (CIs). Descriptive statistics described the distribution of variables in the population. Differences between groups were evaluated using the chi-squared test for categorical variables. Multivariate analyses were conducted in R using the survival Analysis package (v0.3.0). Receiver operating characteristic (ROC) curves were analyzed using the ROCit R package (v2.1.1). All analyses were performed in R (v4.3.2). All statistical tests were two-sided, with $p < 0.05$ indicating statistical significance.

Cell Reports Medicine, Volume *5*

Supplemental information

Analysis of PD1, LAG3, TIGIT, and TIM3 expression in human lung adenocarcinoma reveals a 25-gene signature predicting immunotherapy response

Jean-Philippe Guégan, Florent Peyraud, Bérengère Dadone-Montaudie, Diego Teyssonneau, Lola-Jade Palmieri, Emma Clot, Sophie Cousin, Guilhem Roubaud, Mathilde Cabart, Laura Leroy, Coriolan Lebreton, Christophe Rey, Oren Lara, Ophélie Odin, Maxime Brunet, Lucile Vanhersecke, Ezogelin Oflazoglu Gruyters, Ikbel Achour, Leila Belcaid, Sylvestre Le Moulec, Thomas Grellety, Alban Bessede, and Antoine Italiano

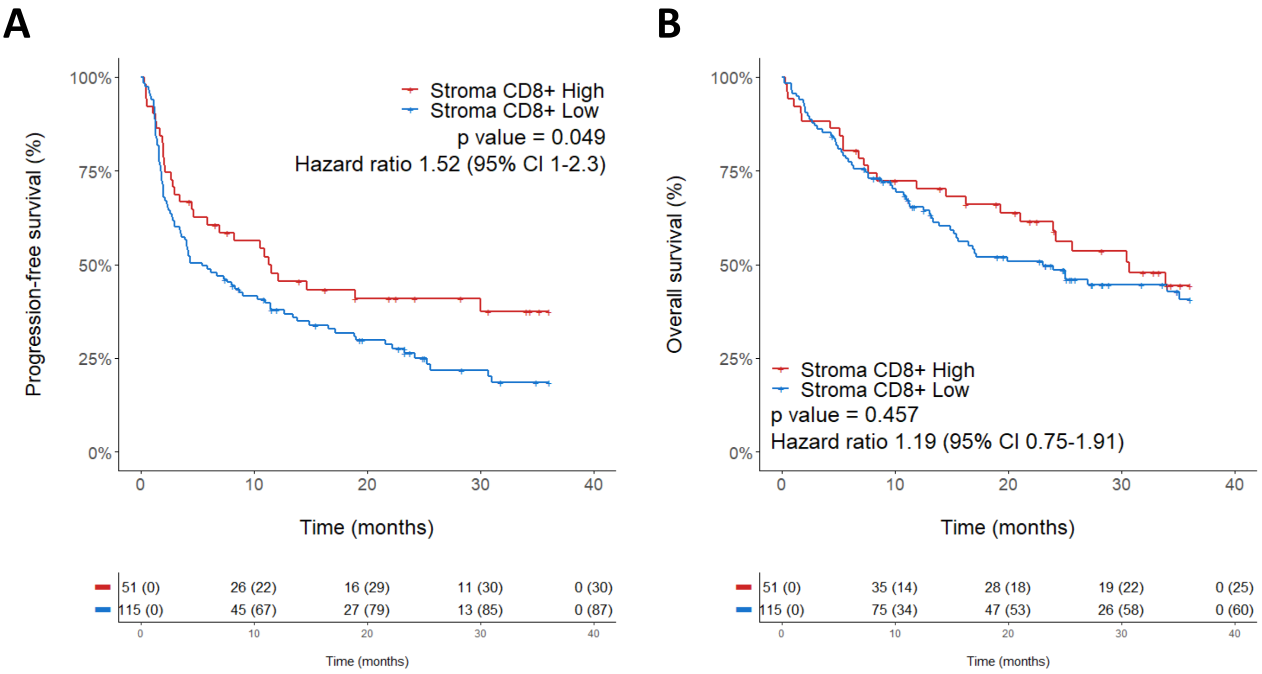

Figure S1. Impact of stromal CD8+ cells on ICI response. Related to Figure 1. Kaplan-Meier curves of progression-free survival (A) and overall survival (B) of LUAD patients with treated with ICI according to level of stroma-infiltrating CD8+ cells. Statistical significance was determined by log-rank test.

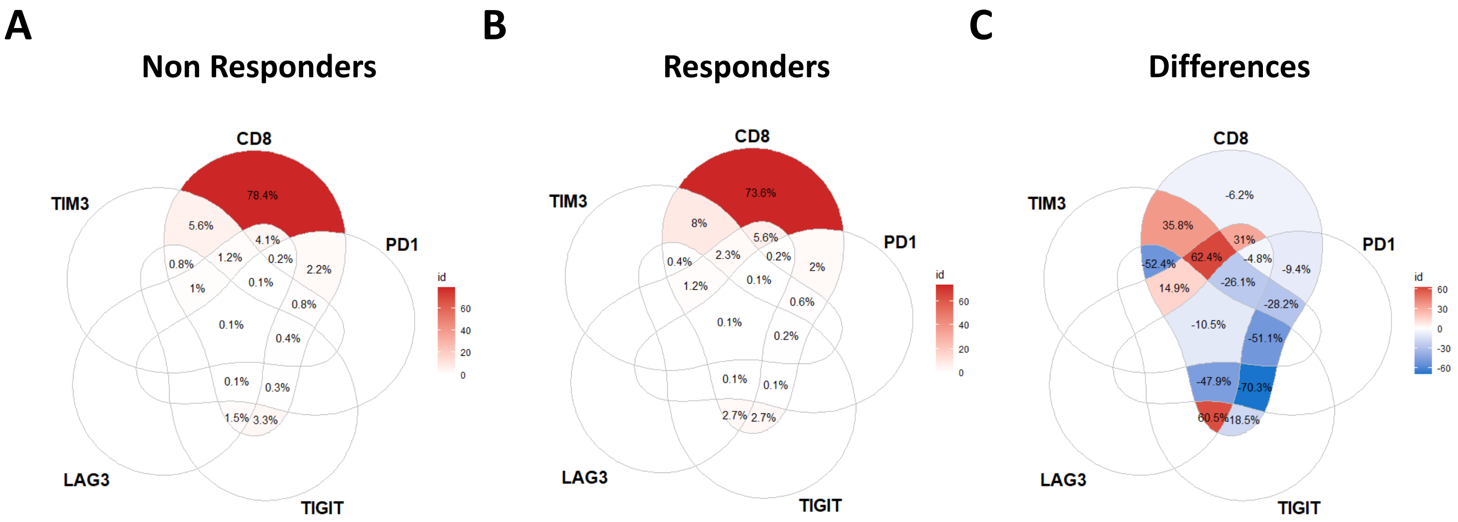

Figure S2. PD1 expression by CD8+ cells correlates with immunotherapy resistance. Related to Figure 1. **(A-C)** Venn diagrams of the expression of exhaustion markers by CD8+ cells from non-responders (A) and responders (B) patients. Differences between responders and non-responders CD8+ cells are displayed in (C). For the analysis, cohort (n=166 patients) was down-sampled to 1000 CD8+ cells per patient. Median of cell distribution are shown.

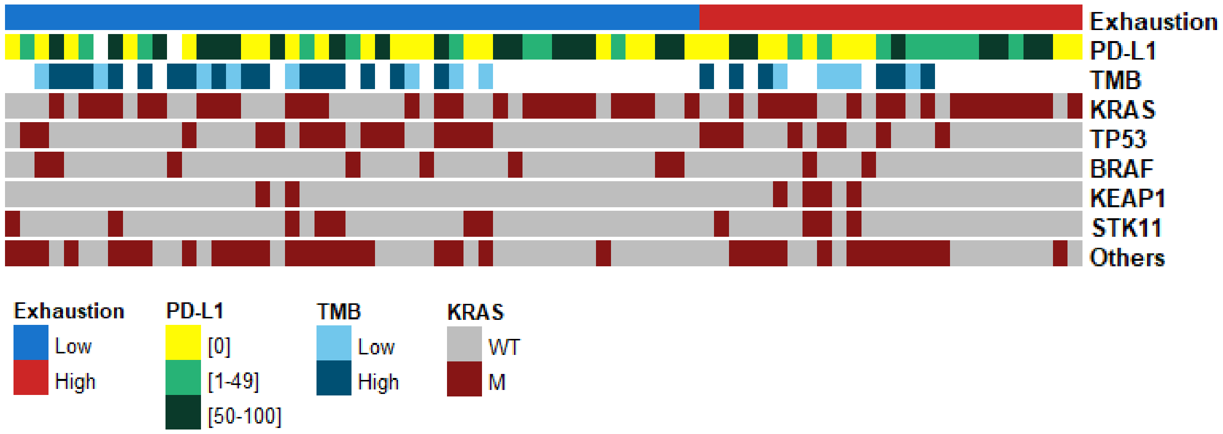

Figure S3. Mutational landscape of LUAD patients treated with ICI according to exhaustion level of CD8+ cells. Related to Figure 1. TMB stands for Tumor Mutational Burden.

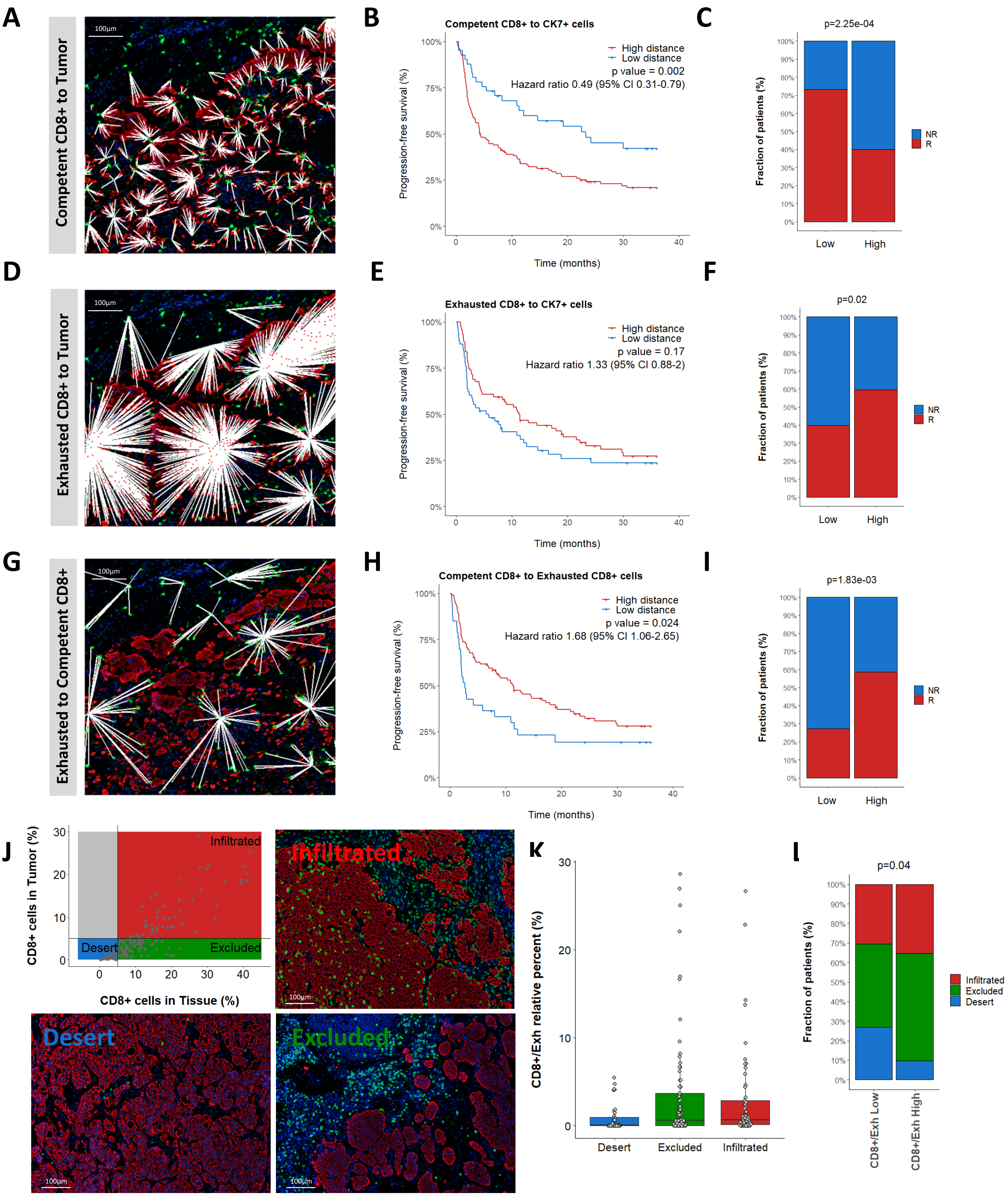

Figure S4. Spatial analysis of the CD8+ distribution in LUAD patients. Related to Figure 1. Intercellular distances were computed between "Competent" CD8+ (CD8+/PD1-), "Exhausted" CD8+ (CD8+/PD1+ ± LAG3/TIGIT/TIM3+) and tumor (CK7+) cells. (**A-D-G**) Illustration of the calculated distances between indicated cells. (**B-E-H**) Kaplan-Meier curves of progression-free survival of LUAD patients classified as "High" or "Low" based on calculated distances. Statistical significance was determined by log-rank test. (**C-F-I**) Histograms of the clinical response achieved in patients with « High » versus « Low » intercellular distances. Statistical significance was determined by chi-squared test. (**J**) Illustration of Immune contexture definition in LUAD patients. A "Desert" phenotype is considered when the sample exhibits less than 5% of CD8+ in the whole section. An "Excluded" phenotype is defined by less than 5% of CD8+ in the tumor areas of the sample. Otherwise, the sample is considered as "Infiltrated" (**K**) Boxplot representation (median ± interquartiles) of CD8+ exhaustion according to immune contexture. (**L**) Histograms of the immune contexture achieved in patients with « High » versus « Low » CD8+ exhaustion. Statistical significance was determined by chi-squared test.

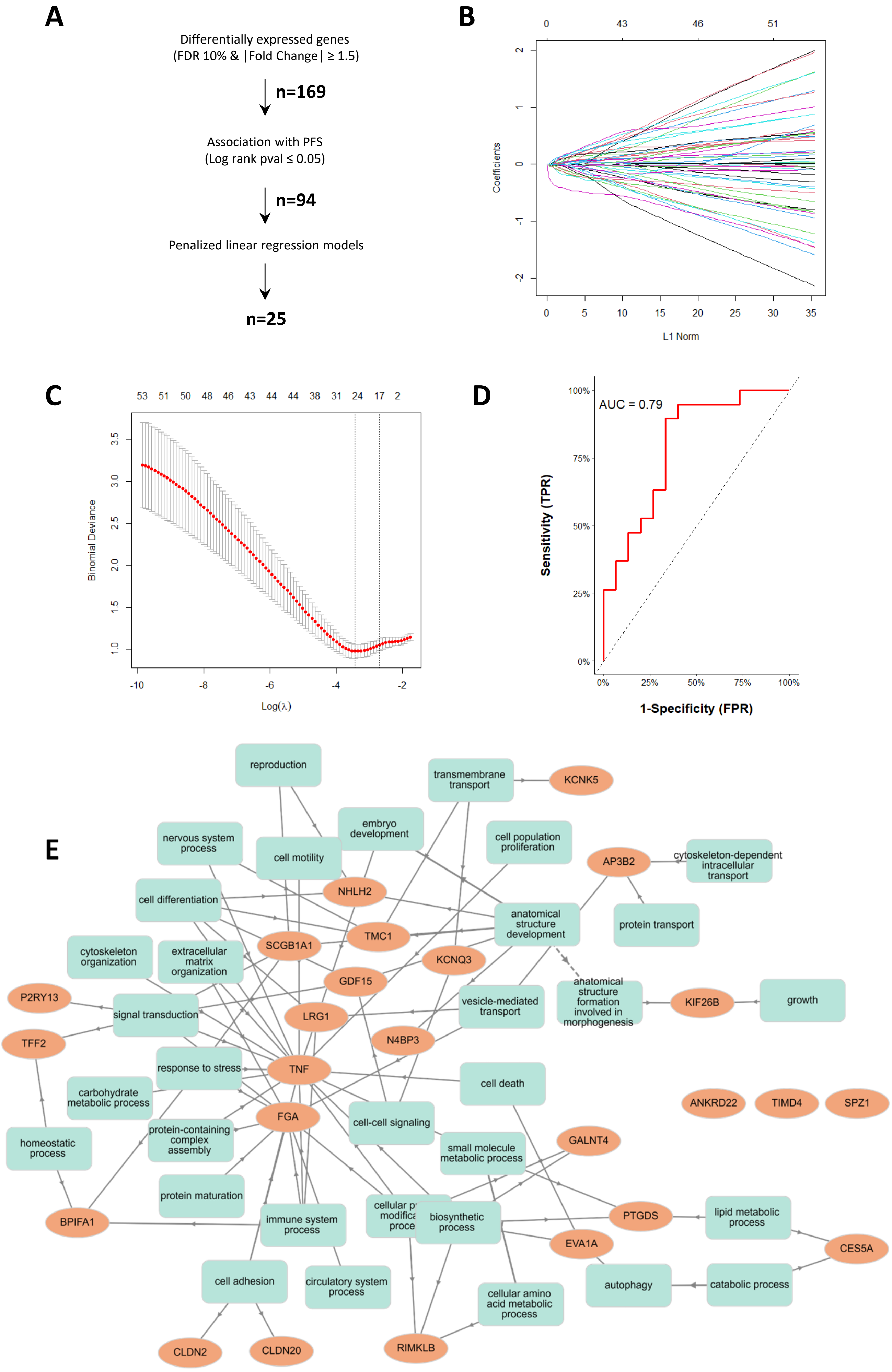

Figure S5. Establishment of a gene signature for the prediction of CD8+ exhaustion. Related to Figure 2. (**A**) Gene signature building strategy (**B**) Coefficient profile plot of the fitted glmnet model (**C**) Cross-validation curve (**D**) ROC curve for the prediction of CD8+ exhaustion in validation cohort (n=35 patients). (**E**) Relationship mapping of signature predictors to Gene Ontology terms.

**Age**	
≤65	89 (54)
>65	77 (46)
**Gender**	
Female	56 (34)
Male	110 (66)
**TPS**	
PD-L1 <1%	64 (39)
PD-L1 1-49%	53 (32)
PD-L1 ≥50%	47 (28)
NA	2 (1)
**ECOG PS**	
0-1	127 (77)
>1	39 (23)
**Immunotherapy**	
Anti-PD1	136 (82)
Anti-PD-L1	23 (14)
Combination**	7 (4)
**Response**	
PD	63 (38)
SD	23 (14)
OR	80 (48)
**Total**	166

*ECOG PS: Eastern Cooperative Oncology Group Performance Status; OR: overall response (partial response or complete response); PD: progressive disease; SD: stable disease; TPS: tumor proportion score.

** Combination of Immunotherapy (Durvalumab-Tremelimumab, Pembrolizumab-Feladilimab, Atzeolizumab-Bevacizumab, Atezolizumab-Daratumumab)

Supplementary Table 1. Clinical characteristics of patients. Related to Figure 1

	CD8+/Exh. High	CD8+/Exh. Low
**Age**		
≤65	27 (53)	62 (54)
>65	24 (47)	53 (46)
**Gender**		
Female	18 (35)	38 (33)
Male	33 (65)	77 (67)
**TPS**		
PD-L1 <1%	19 (37)	45 (39)
PD-L1 1-49%	17 (33)	36 (31)
PD-L1 ≥50%	15 (29)	32 (28)
NA	0 (0)	2 (2)
**ECOG PS**		
0-1	35 (69)	92 (80)
>1	16 (31)	23 (20)
**Immunotherapy**		
Anti-PD1	43 (84)	93 (81)
Anti-PD-L1	8 (16)	15 (13)
Combination**	0 (0)	7 (6)
**Response**		
PD	25 (49)	38 (33)
SD	9 (18)	14 (12)
OR	17 (33)	63 (55)
**Total**	51	115

*ECOG PS: Eastern Cooperative Oncology Group Performance Status; OR: overall response (partial response or complete response); PD: progressive disease; SD: stable disease; TPS: tumor proportion score.

** Combination of Immunotherapy (Durvalumab-Tremelimumab, Pembrolizumab-Feladilimab, Atzeolizumab-Bevacizumab, Atezolizumab-Daratumumab)

Supplementary Table 2. Clinical characteristics of patients according to exhaustion levels. Related to Figure 1

	DESeq2			PFS log-Rank	Risk Score
	**LogFC**	**pvalue**	**padj**	**pvalue**	**Coefficient**
RIMKLB	1.636	5.00E-09	3.95E-05	2.50E-04	-0.101
TMC1	-2.964	1.80E-08	6.98E-05	3.07E-02	0.148
BPIFA1	-2.088	7.09E-07	1.32E-03	2.47E-02	0.032
SCGB1A1	2.701	7.48E-07	1.32E-03	9.49E-05	-0.042
FGA	2.189	5.74E-06	4.96E-03	2.73E-05	0.022
CLDN2	-2.274	2.84E-05	1.41E-02	6.30E-04	0.044
CES5A	2.502	3.92E-05	1.76E-02	6.42E-04	-0.115
GALNT4	1.015	5.02E-05	1.98E-02	1.35E-03	-0.007
AP3B2	-2.091	6.86E-05	2.40E-02	3.26E-02	0.161
GDF15	-1.039	7.27E-05	2.47E-02	3.60E-02	0.168
CLDN20	3.168	1.06E-04	2.87E-02	1.34E-02	-0.151
KCNK5	-1.008	1.00E-04	2.87E-02	4.42E-02	0.051
TFF2	2.087	1.16E-04	3.00E-02	4.22E-03	-0.022
EVA1A	-1.014	2.72E-04	4.85E-02	2.98E-02	0.076
SPZ1	3.207	2.99E-04	4.98E-02	4.19E-03	-0.002
NHLH2	-1.864	3.44E-04	5.33E-02	5.66E-03	0.102
N4BP3	-1.000	3.72E-04	5.50E-02	4.01E-03	0.138
ANKRD22	0.791	3.76E-04	5.52E-02	1.21E-02	-0.431
P2RY13	0.707	4.05E-04	5.66E-02	1.45E-04	-0.052
LRG1	1.036	4.57E-04	6.10E-02	2.78E-02	0.027
PTGDS	0.946	5.03E-04	6.40E-02	3.71E-02	-0.188
TNF	0.968	5.03E-04	6.40E-02	4.78E-02	-0.040
KCNQ3	-1.014	5.24E-04	6.52E-02	2.53E-02	0.001
TIMD4	1.291	7.22E-04	7.85E-02	9.20E-06	-0.147
KIF26B	-0.697	1.01E-03	9.42E-02	4.02E-02	0.113

Supplementary Table 3. CD8+ exhaustion gene signature - 25 genes. Related to Figure 2
